# Static and dynamic balance in children and adolescents with autism spectrum disorder compared with typically developing peers: a systematic review and meta-analysis

**DOI:** 10.1007/s00431-026-06871-0

**Published:** 2026-03-24

**Authors:** Paloma Martín-Díaz, María Carratalá-Tejada, Victor Navarro-López, Pilar Fernández-González, Alicia Cuesta-Gómez

**Affiliations:** 1 International PhD School, Rey Juan Carlos University, 28008 Madrid, Spain; 2 Department of Physical Therapy, Occupational Therapy, Rehabilitation and Physical Medicine, Faculty of Health Sciences, Rey Juan Carlos University, 28922 Madrid, Spain

**Keywords:** Static balance, Dynamic balance, Assessment, Autism spectrum disorder and postural control

## Abstract

Children and adolescents with autism spectrum disorder (ASD) often present motor difficulties, particularly in postural control, which can affect their autonomy and participation in daily activities. Despite growing interest in balance assessment, there is no comprehensive synthesis comparing balance performance in ASD using both observational and instrumental methods. To compare the static and dynamic balance of children and adolescents with ASD versus typically developing (TD) peers, and to identify the tools most used to assess balance in this population. A systematic review and meta-analysis were conducted following PRISMA guidelines. Six electronic databases were searched to November 2025. Inclusion criteria focused on studies assessing static or dynamic balance in children aged 6–18 with ASD, using validated observational or instrumental tools, and including a TD group. Data extraction, methodological quality assessment, risk‑of‑bias evaluation, and GRADE assessment were performed independently by two reviewers, with discrepancies resolved by discussion or by a third reviewer. Standardized mean differences (SMDs) were calculated using random-effects models, established a priori to account for expected clinical heterogeneity. A total of 34 studies were included in the descriptive synthesis and 16 in the meta-analyses, encompassing 1278 participants (612 ASD, 666 TD). Observational tools (e.g., MABC-2, BOT-2) revealed significantly poorer balance in ASD participants ((SMD = –0.66; 95% CI: –1.07 to –0.25; p = 0.002). Force platform assessments demonstrated larger differences across postural sway measures: mediolateral displacement (stable surface: eyes open SMD = 0.83, eyes closed SMD = 0.56), anteroposterior displacement (stable surface: eyes open SMD = 0.97, eyes closed SMD = 0.27), COM displacement area (SMD = 1.15–7.72 depending on condition), and COM velocity (SMD = 1.00–3.23 depending on condition). Heterogeneity ranged from moderate to very high (I^2^ = 0–98%). Sensitivity analyses indicated that some effect estimates, particularly for COM displacement area, were influenced by individual studies. The overall certainty of evidence was low to very low according to GRADE.

*Conclusion*: Children with ASD may exhibit poorer static and dynamic balance compared to TD peers using both observational and instrumental assessments. Further high-quality studies are needed to strengthen the evidence base and enhance ecological validity in real-world settings.

**Table:** 

**What is Known:** • *Children and adolescents with autism spectrum disorder (ASD) frequently experience balance problems that affect daily functioning and participation.* • *Earlier reviews have described postural control in ASD but have not examined in depth the instruments used for its assessment.*
**What is New:** • *This review provides a systematic overview of the clinical and biomechanical tools applied to evaluate balance in ASD.* • *It also compares the performance of children across different assessment methods, offering practical guidance for clinicians and educators when selecting appropriate measures.*

**Supplementary Information:**

The online version contains supplementary material available at 10.1007/s00431-026-06871-0.

## Introduction

Autism spectrum disorder (ASD) involves social, communicative, behavioral, and motor difficulties. [1–4] Children with ASD often show delayed motor skills, abnormal muscle tone, coordination deficits, atypical gait, and impaired static and dynamic balance, affecting daily functioning. [5, 6] Postural control (maintaining stability and orientation of the body in space) [7] is frequently compromised, influencing sensorimotor and social development. [8] Balance can be assessed under static conditions (maintaining posture) [9] or dynamic conditions (stability during movement), [10] capturing complementary aspects of postural control.

Studies using clinical scales, posturography, and 3D motion capture consistently show that children with ASD often have impaired sensory processing, [11–15] leading to increased sway, higher fall risk, [3, 16] and restrictions in physical and recreational participation. [14, 17] Observational tools are practical for clinical use, whereas instrumental methods provide objective, quantitative data but need specialized equipment.

Previous reviews [6, 15, 18] highlighted balance difficulties in ASD but did not focus on assessment methods or certainty of evidence, nor distinguish between clinical and instrumental approaches. This systematic review and meta-analysis address these gaps by synthesizing evidence on balance control in youth with ASD versus typically developing (TD) peers, examining assessment tools, and grading evidence quality using GRADE to guide professionals in selecting appropriate measures. Accurate evaluation of static and dynamic balance is essential for supporting functional mobility and quality of life in this population. [14]

This systematic review and meta-analysis (1) synthesizes evidence on balance control in youth with ASD versus TD peers, (2) examines assessment tools, and (3) evaluates evidence quality using GRADE, aiming to guide professionals in selecting appropriate tools to assess balance deficits.

## Material and methods

### Design

This review followed PRISMA guidelines for systematic reviews [19] and was registered in PROSPERO (CRD42023438863). All steps of the review process (literature search, title/abstract screening, full-text assessment, data extraction, methodological assessment and GRADE assessment) were performed independently by two reviewers, and any discrepancies were resolved through discussion or adjudication by a third reviewer.

### Search strategy and databases

The following databases were searched through November 2025: PubMed, Web of Science, Embase, Cochrane, PEDro, and Scielo. The complete search strategy is presented in online supplementary material (OSM). (Table 1).

### Screening process and eligibility criteria

Screening was conducted in two phases: title/abstract review and full-text assessment. Two researchers (P.M.D. and V.L.N.) independently and blindly evaluated records using predefined criteria, with disagreements resolved by a third reviewer (P.F.G.).

Inclusion criteria: (1) observational studies or clinical trials; (2) English or Spanish publications; (3) comparison of static or dynamic balance in children/adolescents with ASD versus TD peers; (4) participants aged 6–18 years; (5) balance assessed via validated scales or posturography. Exclusion criteria: (1) outcomes of interest not reported; (2) comorbid conditions; (3) intervention studies included only for baseline data; (4) observational studies without controls excluded. For mixed-condition studies, only ASD participants and their matched controls were analyzed.

### Data extraction

A standardized method was used to extract data from eligible studies. Information included first author, year, design, sample size, demographics, instrumental analysis, outcomes, and means ± SD. When data were unclear, authors were contacted; if no response was received, the data were excluded.

### Methodological quality

The methodological quality of quantitative studies was evaluated using the McMaster Critical Review Form for Quantitative Studies, [20] which comprises 15 items. Each item was rated as ‘yes’ (criterion met), ‘no’ (criterion not met), or ‘not addressed.’ Two reviewers independently applied the checklist to each study. As part of the scoring procedure, items marked as N/A (Not Applicable) were excluded from the denominator, as they did not apply to the study design being assessed, whereas all items rated as Yes, No, or Not addressed were counted toward the total number of applicable items. This approach ensured that each study was evaluated according to the methodological criteria relevant to its specific design. Higher scores indicated superior methodological quality. Disagreements were resolved by discussion or a third reviewer.

### Data synthesis and analysis

Quantitative analysis compared youth with ASD and TD peers on static and dynamic balance using baseline means and SDs. Standardized mean differences (SMDs), with 95% confidence intervals (CIs) were calculated using random-effects models to account for heterogeneity. SMDs were classified as large (> 0.8), medium (0.5–0.8), or small (0.2–0.5); p < 0.05 was considered significant.

Between-study heterogeneity was assessed using Cochran’s Q test (p < 0.05) [21] and the inconsistency index (I^2^), with I^2^ values > 25%, > 50%, and > 75% indicating low, moderate, and high heterogeneity, respectively. [22] Complementary to Q, I^2^ has limited power when few studies are included. [22] Separate meta-analyses were conducted for each instrumental condition (eyes open/closed; stable/unstable) to maintain effect size independence, and subgroup analyses by ASD severity and age were performed when data allowed.

When full texts were unavailable, authors were contacted up to three times; non-responding studies were excluded. Data were extracted as reported or digitized from graphs when necessary. Studies lacking data were excluded.

Funnel plots were used to visually assess potential publication bias and were interpreted cautiously when fewer than 10 studies were available. Analyses were conducted in Review Manager 5.3.

Evidence certainty was assessed using GRADE. Risk of bias was downgraded for methodological limitations (McMaster tool), inconsistency for high heterogeneity (I^2^ > 50–75%) or unstable sensitivity analyses, and imprecision for wide confidence intervals or small sample sizes. Publication bias was considered when funnel plots were asymmetric. No downgrading for indirectness was applied, as populations and outcomes matched the prespecified PICO.

## Results

### Study selection

**Fig. 1:**
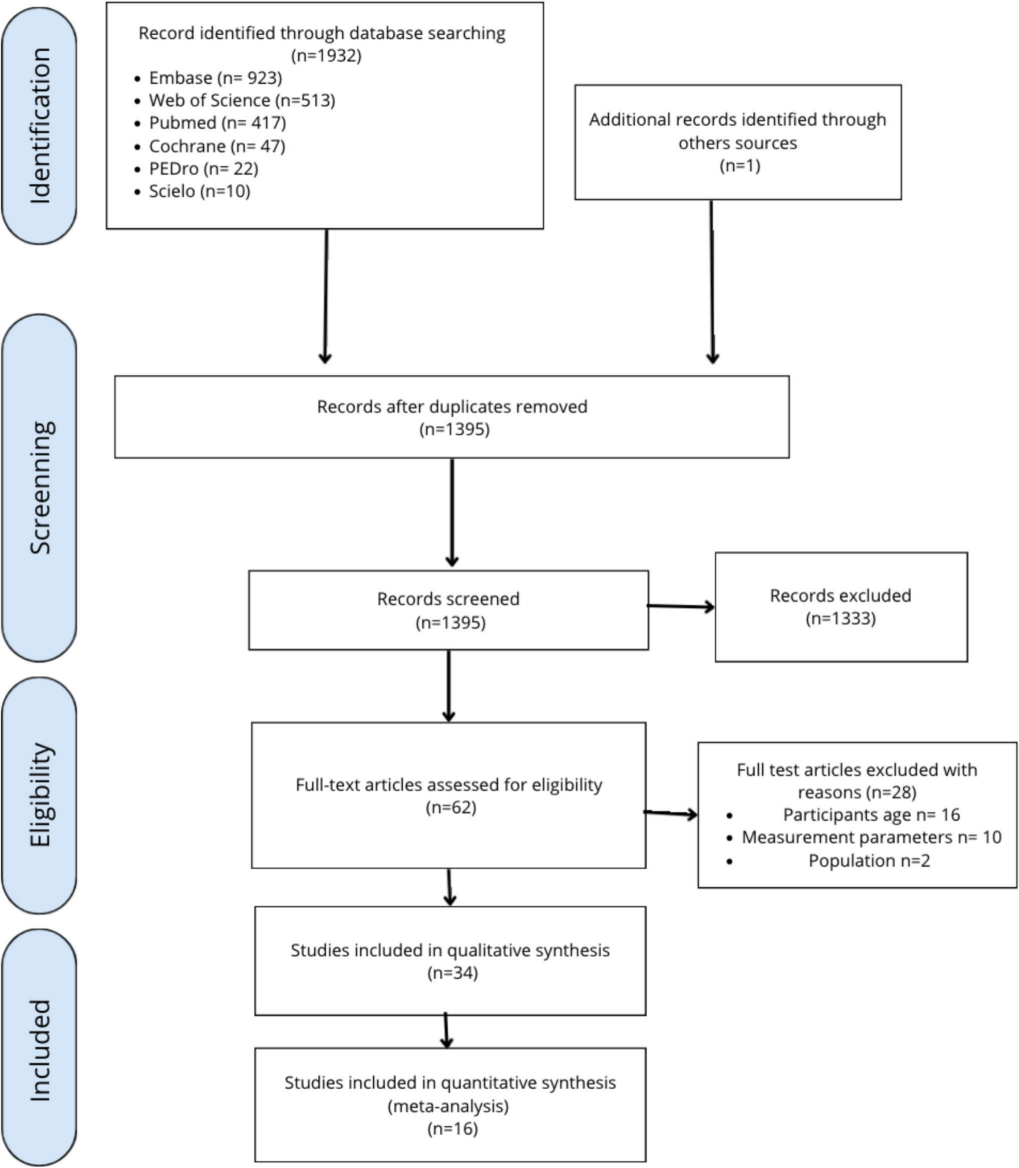
PRISMA flow diagram

A total of 1932 records were identified: (PubMed 417; Web of Science 513; Embase 923; Cochrane 47; PEDro 22; Scielo 10), plus one from other sources. After removing duplicates, 1395 records were screened, and 1333 were excluded. Sixty-two full texts were assessed; 28 were excluded (see OSM, Table 2). Finally, 34 studies were included in the descriptive synthesis and 16 in the meta-analysis. The selection process is presented in the PRISMA flow diagram (Fig. 1).

### Characteristics of included studies

Table 3 (OSM) summarizes characteristics of the included studies. Thirty-four studies were included in the descriptive synthesis (31 observational and 3 experimental). A total of 1278 participants were included: 612 with ASD and 666 TD. Participants in the ASD group had a weighted mean age of 10.68 years, similar to the TD group (10.67 years). Gender data were available for 557 ASD and 638 TD participants; five studies did not report this information. [23–27] In the ASD group, 94.8% were male on average, compared to 70.4% in the TD group. Twenty-nine studies used instrumental tools, [11, 12, 14, 24–48] while 11 employed observational assessments. [14, 26, 28, 32, 34, 38, 44, 49, 51–53]

### Methodological quality

Methodological quality assessed with the McMaster Critical Review Form was generally acceptable. N/A items were excluded, while Yes, No, or Not addressed counted toward the total, ensuring evaluation relevant to each study’s design. Most studies scored 9–11. Highest-quality studies were Surgent et al. [44], (13/14) and Abdel-Ghafar et al. [29] and Ghanouni et al. [25] (11/13). Lowest scores were 9/13, [28, 30–34, 36, 37, 50, 52] 10/14 [48] and 9/14, [26] mainly due to unaddressed items and limited reporting on sample size justification, clinical importance, and participant dropouts. Full details are in Table 4 (OSM).

### Main descriptive synthesis results

#### Balance performance in observational tools

MABC was used in six studies. Two studies reported poorer balance in the ASD group than in TD peers, [34, 52] while three found no significant differences. [49, 51, 53] Gouleme et al. [32] only reported MABC scores for the ASD group, which prevented comparison with controls.

In BOT-2 studies, the ASD group scored significantly lower than in TD peers; Ardalan et al. [28] observed differences in percentiles, while Kaur et al. [35] and Martin-Diaz et al. [14] reported poorer gross and fine motor performance across various ages and functioning levels. However, Smoot Reinert et al. [26] found similar outcomes between groups.

Finally, one study observed that a lower total score in TGMD-3 was associated with a higher postural sway area on a solid surface but not on an unstable surface. [38]

#### Balance performance in instrumental tools

Nintendo Wii Balance Board studies [11, 28, 33, 43, 49, 51] reported greater postural instability and movement variability in youth with ASD versus TD peers. Ardalan et al. [28] and Graham et al. [33] found increased deficits, particularly under challenging conditions. Visual feedback improved performance, especially in lower-IQ participants, though deficits persisted. [48] Stins et al. [49] showed greater visual dependence, with instability when eyes were closed. Surgent et al. [43] reported improved control and reduced symptoms after biofeedback training. Travers et al. [11] linked lower IQ to greater impairments, highlighting cognitive influences on postural control.

Five studies used the AccuGait force platform. [26, 30, 37, 44, 47] Bojanek et al. [30] reported reduced motor coordination, linking motor rigidity in ASD to balance deficits and repetitive behaviours. Lim et al. [37] found comparable postural stability and attentional demands in ASD and TD children under static visual and non-visual conditions. Stania et al. [26] observed greater anteroposterior sway and lower postural complexity in ASD, with increased sway before and after locomotor transitions. Other studies [44, 47] associated instability, higher COP variability, reduced motor independence, and anticipatory adjustments with repetitive behaviours.

Six studies used the Bertec (Columbus, OH) force platform. [24–26, 39, 40, 46] Fournier et al. [24] reported greater sway and COP–center of mass (COM) distance in ASD during quiet standing, with similar gait initiation. Fradet et al. [46] found increased sway, especially without vision, suggesting visual dependence. Ghanouni et al. [25] observed greater mediolateral sway to social stimuli, linked to autistic traits. Memari et al. [40] found higher sway in both directions, associated with symptom severity. According to Memari et al. the ASD group exhibited significantly more sway overall, with visual activities generating more sway than auditory ones. [39] Smoot Reinert et al. [26] noted temporary postural improvements after vestibular stimulation, supporting targeted sensory interventions.

Five studies used other force platforms. [27, 35, 36, 38, 41] Pettinato et al. [27] reported reduced stability and COP complexity in ASD, linked to repetitive behaviours and balance performance. Li et al. [35] found reduced mediolateral complexity. Lidstone et al. [36] observed greater sway area and mediolateral displacement in ASD. Mache and Todd [38] identified higher sway and poorer motor performance. Miller et al. [41] found similar motor control deficits in developmental coordination disorder (DCD) and ASD, but differences in dynamic balance and movement fluidity.

Abdel-Ghafar et al., using the Modified Clinical Test of Sensory Integration and Balance, found that children with ASD had significantly greater postural sway than in TD peers in all conditions, particularly when visual and somatosensory inputs were disrupted (p < 0.05), indicating sensory-related static postural control deficits. [29]

Three studies used Sensory Organization Test (SOT). [12, 31, 45] All reported reduced stability in ASD, particularly under sensory-challenging conditions. Fears et al. found greater sway variability across conditions despite similar age-related trends. [31] Shabana et al. reported lower SOT scores in ASD, especially with altered somatosensory input, and balance worsened with increasing autism severity. [12] Zoccante et al. observed the greatest deficits in ASD, followed by ADHD, whereas children with Tourette syndrome resembled TD peers, supporting a neurodevelopmental gradient. [45]

### Main quantitative results

#### Balance performance in ASD vs. TD children: assessment using the MABC

**Fig. 2:**
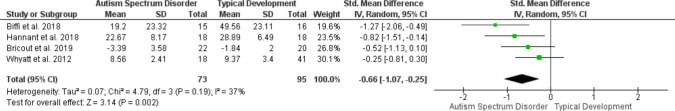
MABC forest plot

Balance assessed with the MABC was significantly lower in children with ASD than in TD peers (SMD = − 0.66; 95% CI: − 1.07 to − 0.25; n = 168; Z = 3.14; p = 0.002), with moderate heterogeneity (I^2^ = 37%; p = 0.19) (Fig. 2). Funnel plot asymmetry suggests possible selection bias.

Each square represents an individual study’s effect size, with size proportional to its weight in the meta-analysis; horizontal lines = 95% CI. The diamond depicts the pooled effect (random-effects model), and the vertical line marks no effect.

#### Balance performance in ASD vs. TD children: assessment using force-platforms measures

COM-related parameters were analyzed under four conditions: eyes open/stable surface, eyes closed/stable surface, eyes open/unstable surface, and eyes closed/unstable surface. Each parameter (e.g., mediolateral displacement) was examined separately, with conditions treated as subgroups.

### Mediolateral displacement of the COM

**Fig. 3:**
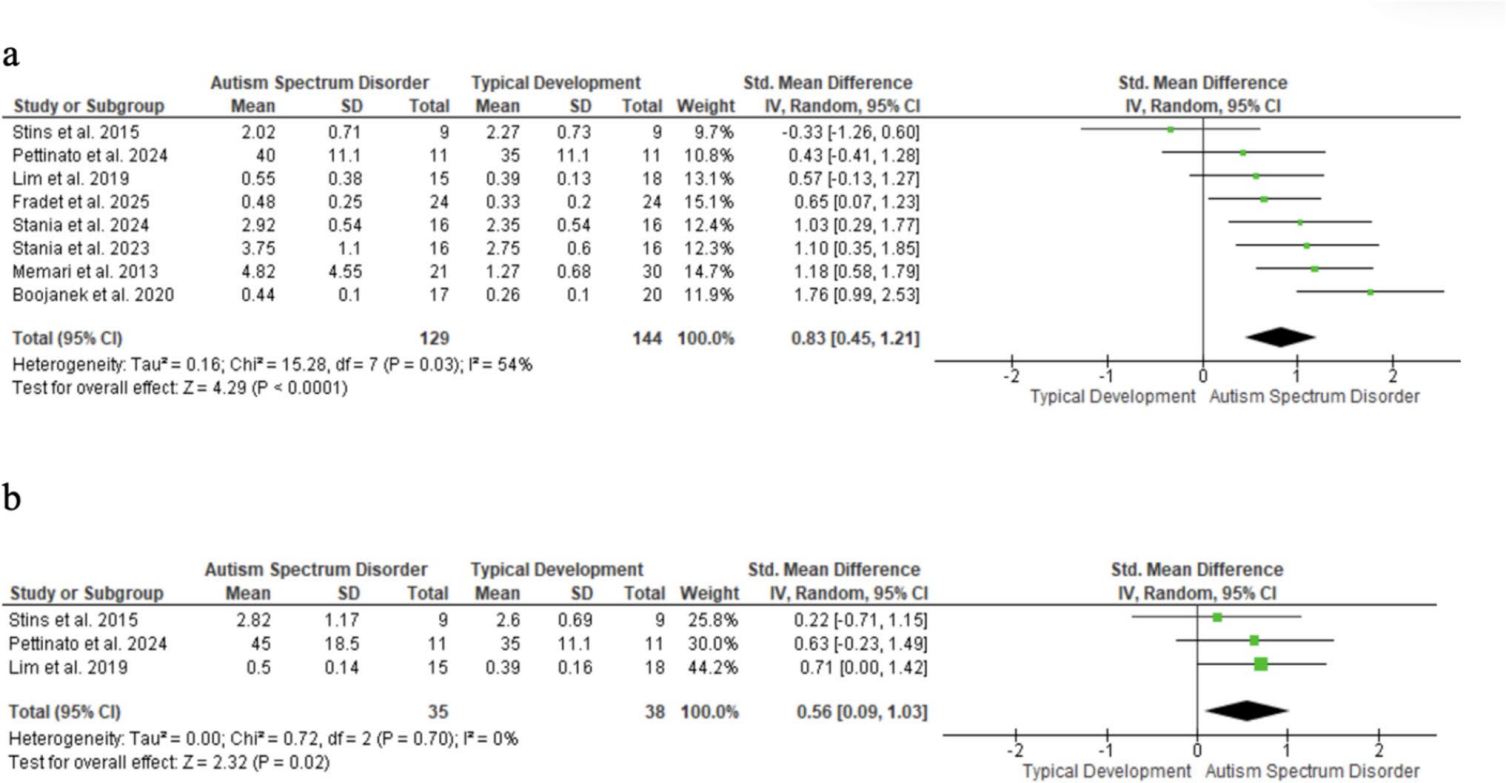
Mediolateral displacement of the COM

Mediolateral COM displacement on stable surfaces was significantly higher in children with ASD than in TD peers, with a large effect in eyes open (SMD = 0.83; 95% CI: 0.45–1.21; n = 273; Z = 4.29; p < 0.001; I^2^ = 54%; p = 0.03) (Fig. 3a) and a moderate effect in eyes closed (SMD = 0.56; 95% CI: 0.09 to 1.03; n = 73; Z = 2.32; p = 0.02) (Fig. 3b). Funnel plot asymmetry suggests possible selection bias.

Each square represents an individual study’s effect size, with size proportional to its weight in the meta-analysis; horizontal lines = 95% CI. The diamond shows the pooled effect (random-effects model), and the vertical line marks no effect. (a) Eyes open; (b) Eyes closed.

### Anteroposterior displacement of the COM

**Fig. 4:**
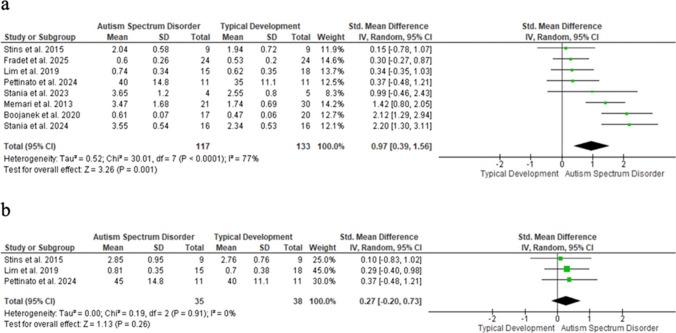
Forest plot of anteroposterior COM displacement

Anteroposterior COM displacement was significantly greater in children with ASD than in TD peers under stable, eyes-open conditions (SMD = 0.97; 95% CI: 0.39–1.56; n = 250; Z = 3.26; p = 0.001), with high heterogeneity (I^2^ = 77%; p < 0.001) (Fig. 4a). The eyes-closed condition was not significant (SMD = 0.27; 95% CI: − 0.20—0.73; n = 73; Z = 1.13; p = 0.26) (Fig. 4b). Funnel plot asymmetry suggests possible publication bias.

Each square represents an individual study’s effect size, with its size proportional to its weight in the meta-analysis; horizontal lines = 95% CI. The diamond represents the pooled effect from a random-effects model, and the vertical line indicates no effect. (a) Eyes open; (b) Eyes closed.

### COM displacement area

**Fig. 5:**
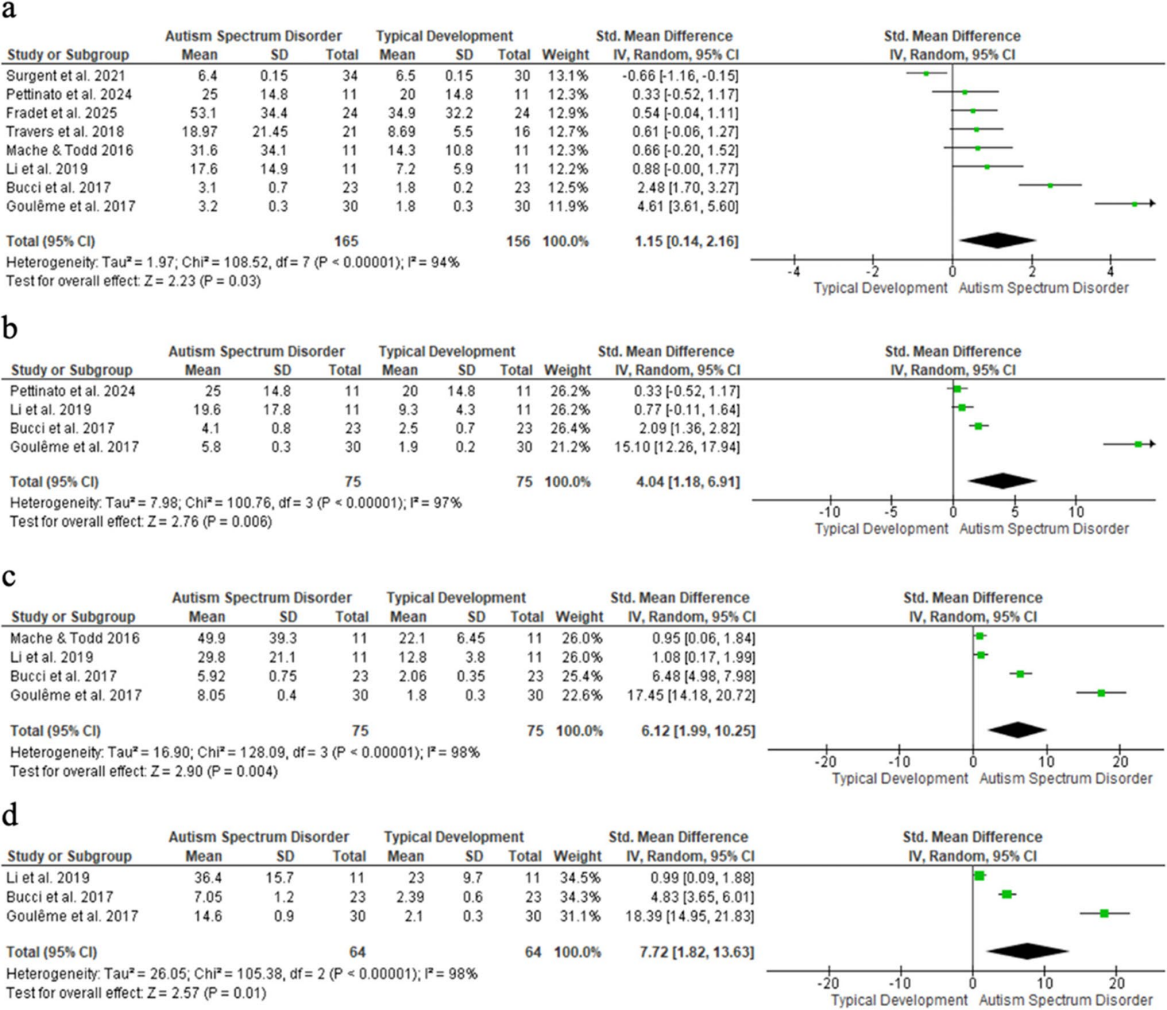
Displacement area of the COM forest plot

COM displacement area was significantly greater in children with ASD than in TD peers, with large effects in all conditions: eyes open–stable (SMD = 1.15; 95% CI: 0.14–2.16; n = 321; p = 0.03; I^2^ = 94%); eyes closed–stable (SMD = 4.04; 95% CI: 1.18–6.91; n = 150; p = 0.006; I^2^ = 97%); eyes open–unstable (SMD = 6.12; 95% CI: 1.99–10.25; n = 150; p = 0.004; I^2^ = 98%); and eyes closed–unstable (SMD = 7.72; 95% CI: 1.82–13.63; n = 128; p = 0.01; I^2^ = 98%) (Fig. 5). Funnel plot asymmetry suggests possible selection bias.

Each square shows an individual study’s effect size, sized by study weight; horizontal lines = 95% CI. The diamond = pooled effect; vertical line = no effect. (a) Eyes open, stable; (b) Eyes closed, stable; (c) Eyes open, unstable; (d) Eyes closed, unstable.

A sensitivity analysis excluded the study by Gouleme et al. [32] due to its potential outlier effect. This removal eliminated statistical significance and reduced effect sizes across all conditions, suggesting overestimation when included: eyes open, stable surface (SMD = 0.67; 95% CI: − 0.07 to 1.40; n = 261; p = 0.07; I^2^ = 87%), eyes closed, stable surface (SMD = 1.08; 95% CI: − 0.01 to 2.17; n = 90; p = 0.05; I^2^ = 81%), eyes open, unstable surface (SMD = 2.77; 95% CI: − 0.07 to 5.61; n = 90; p = 0.06; I^2^ = 95%), and eyes closed, unstable surface (SMD = 2.89; 95% CI: − 0.88 to 6.65; n = 68; p = 0.13; I^2^ = 96%).

### Velocity of COM displacement

**Fig. 6:**
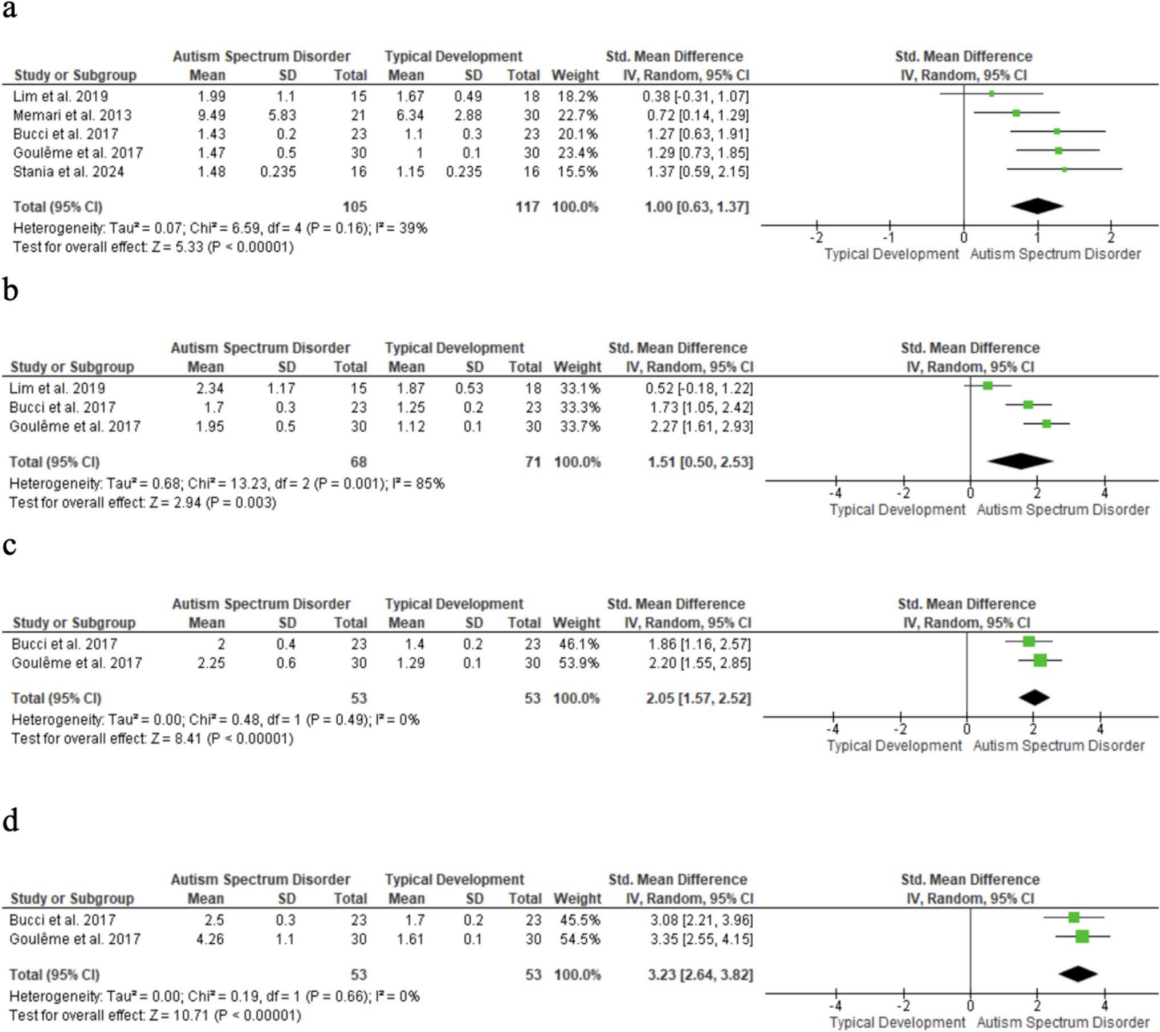
Velocity displacement of the COM forest plot

COM displacement velocity measured by force platforms was significantly higher in children with ASD than in TD peers, with large effects across all conditions: eyes open, stable (SMD = 1.00; 95% CI: 0.63–1.37; n = 222; p < 0.001; I^2^ = 39%); eyes closed, stable (SMD = 1.51; 95% CI: 0.50–2.53; n = 139; p = 0.003; I^2^ = 85%); eyes open, unstable (SMD = 2.05; 95% CI: 1.57–2.52; n = 106; p < 0.001; I^2^ = 0%); and eyes closed, unstable (SMD = 3.23; 95% CI: 2.64–3.82; n = 106; p < 0.001; I^2^ = 0%) (Fig. 6). Funnel plot asymmetry suggests potential selection bias.

Squares show individual study effect sizes (size reflects weight); horizontal lines indicate 95% CIs. The diamond represents the random-effects pooled estimate, and the vertical line indicates no effect. (a) Eyes open, stable; (b) Eyes closed, stable; (c) Eyes open, unstable; (d) Eyes closed, unstable.

### Quality of evidence

Table 5 shows the GRADE evaluation. Overall evidence quality was very low to low. Limited study numbers, methodological flaws, high heterogeneity, and potential publication or selection bias reduced certainty despite moderate-to-large effect sizes. Unstable sensitivity analyses further lowered confidence in pooled estimates.

## Discussion

This systematic review and meta-analysis examined static and dynamic balance in children and adolescents with ASD versus TD peers using observational and instrumental tools. Despite methodological variability, samples were broadly comparable. Pooled results suggest associations between ASD and poorer postural control across conditions. However, given the high risk of bias and low to very low certainty of evidence, these findings should be interpreted with caution and cannot be taken to imply causal differences in postural control.

Observational findings were inconsistent. Two MABC studies showed TD advantages on the balance subscale. [34, 52] Odeh et al. [54] found lower balance scores in ASD on the MABC-2 and BOT-2, whereas three studies found no differences [49, 51, 53] questioning sensitivity across the spectrum. Variations in age, IQ, functional level, and scoring may explain discrepancies. Gouleme et al. [32] included only ASD data, precluding TD comparison.

BOT-2 studies reported significant group differences. [14, 26, 28, 50] Kaur et al. reported HASD–LASD differences and associations with IQ. [50] Similarly, our group identified significant ASD–TD differences on the BOT-2 short form. [14] In contrast, Smoot Reinert et al. found no differences, [26] likely due to the small sample, highlighting how sample size affects detection of motor or balance differences.

Preliminary evidence indicates significant balance impairments in ASD compared with TD. Meta-analysis of the MABC balance subscale showed a moderate effect favoring TD (SMD = − 0.66; I^2^ = 37%), [29, 51–53] consistent with reports of postural deficits in ASD. [28, 32, 38] However, funnel plot asymmetry suggests publication or selection bias, warranting cautious interpretation.

Force-platform findings were consistent with observational results, indicating poorer balance and greater sway in ASD. Most studies reported deficits, particularly under multisensory or cognitively demanding conditions. [23–25, 28–33, 36]

Mediolateral COM displacement showed large (eyes open, SMD = 0.83) and moderate (eyes closed, SMD = 0.56) effects [27, 30, 37, 40, 42, 47, 49] particularly in stable, eyes-open conditions, aligning with Lidstone et al. [36] Lim et al. attributed null findings in younger samples to developmental variability. [37]

Anteroposterior displacement showed a large effect (SMD = 0.97; I^2^ = 70%), [26, 27, 30, 37, 40, 47, 49] indicating multidirectional postural instability in ASD. Notably, only the eyes-open condition reached significance.

COM area showed large effect sizes in eyes open–stable, eyes closed–stable, and eyes closed–unstable conditions [11, 23, 32, 35, 38, 43] supporting multisensory integration deficits, [55] but with high heterogeneity and potential bias (e.g., [32]).

COM velocity also showed strong effects across conditions. [23, 32, 37, 40, 47] This suggests that different sensory conditions differentially affect balance control in ASD. However, very low GRADE certainty and high heterogeneity warrant cautious interpretation.

Both methods identify balance differences in ASD, but scales such as the MABC-2 and BOT-2 provide greater ecological validity for daily-life interventions, as they assess balance and coordination in functional contexts. [54]

Clinically, balance assessment is important because motor difficulties can affect participation and daily functioning. Therefore, integrating standardized balance assessments is essential to support targeted early interventions.

### Study limitations

Several limitations should be noted. Assessment tools and protocols varied, and observational and instrumental measures likely captured partially different constructs, limiting comparability. Variations in visual input, surface, stance, trial duration, repetitions, and task complexity increased heterogeneity. Although SMDs standardized outcomes, pooling measures from different frameworks may have introduced residual conceptual heterogeneity. Participant characteristics (age, cognitive level, ASD severity, comorbidities) were inconsistently reported or controlled. Most studies were observational with moderate-to-high risk of bias, limiting causal inference. Restricting the search to English and Spanish may have introduced language bias, and some meta-analyses included fewer than 10 studies, limiting publication bias assessment. Additionally, several studies contributed multiple effect sizes from the same samples; although analyzed as separate conditions for conceptual comparability, statistical dependence may not have been fully addressed. Despite independent extraction and bias assessment, minor errors may remain. Overall, these factors resulted in low or very low GRADE certainty and warrant cautious interpretation.

## Conclusion

This meta-analysis suggests that children with ASD may show poorer balance than TD peers in static and dynamic tasks. Observational tools indicated moderate motor differences, and force-platform measures suggested larger differences in sway and center-of-mass parameters. However, because the certainty of evidence was low to very low these findings should be interpreted cautiously.

## Supplementary Information

Below is the link to the electronic supplementary material.

ESM 1 DOCX (84.1 KB)

## Data Availability

No datasets were generated or analysed during the current study.

## Abbreviations

ADOS-2: Autism Diagnostic Observation Schedule, 2nd edition
APA: Anticipatory Postural Adjustments
ASD: Autism Spectrum Disorder
BMI: Body Mass Index
BOT-2: Bruininks-Oseretsky Test of Motor Proficiency, 2nd edition
CARS: Childhood Autism Rating Scale
CDP: Computerized Dynamic Posturography
COM: Center of Mass
COP: Center of Pressure
DCD: Developmental Coordination Disorder
FSIQ: Full Scale Intelligence Quotient
GRF: Ground Reaction Force
HASD/LASD: High-functioning ASD / Low-functioning ASD
IQ: Intelligence Quotient
MABC/MABC-2: Movement Assessment Battery for Children / 2nd edition
MD: Mean Difference
MI: Movement Integration
OSM: Online Supplemental Material
PANESS: Physical and Neurological Examination for Subtle Signs
PBS: Pediatric Balance Scale
PPVT-R: Peabody Picture Vocabulary Test – Revised
RBS-R: Repetitive Behavior Scale – Revised
RQ: Romberg Quotient
RMS: Root Mean Square
SCQ: Social Communication Questionnaire
SD: Standard Deviation
SMD: Standardized Mean Difference
SOT: Sensory Organization Test
SPL: Sway Path Length
SRS-2: Social Responsiveness Scale, 2nd edition
TD: Typically Developing
TGMD-3: Test of Gross Motor Development, 3rd edition
VO₂ peak: Maximal Oxygen Consumption
WASI: Wechsler Abbreviated Scale of Intelligence
WISC-IV: Wechsler Intelligence Scale for Children, 4th edition
WOS: Web of Science
