# Supplementary material for: Static and dynamic balance in children and adolescents with autism spectrum disorder compared with typically developing peers: a systematic review and meta-analysis
Source: Eur J Pediatr. 2026 Mar 24;185(4):209. doi: 10.1007/s00431-026-06871-0 (PMC13013233; doi:10.1007/s00431-026-06871-0)
Supplement: Supplementary file 1 — DOCX (84.1 KB) [file 431_2026_6871_MOESM1_ESM.docx]

ONLINE SUPPLEMENTARY MATERIAL

**Table 1.** Complete search strategy. (Online supplementary material)

| **Databases** | **Search strategy** | **Studies** |
| --- | --- | --- |
| **Pubmed** | ('autism spectrum disorder' OR 'autism' OR 'autism assessment') AND ('postural balance' OR 'postural stability' OR 'postural control' OR 'posture') | 417 |
| **WOS** | ('autism spectrum disorder' OR 'autism' OR 'autism assessment') AND ('postural balance' OR 'postural stability' OR 'postural control' OR 'posture') | 513 |
| **EMBASE** | ('autism spectrum disorder' OR 'autism' OR 'autism assessment') AND ('postural balance' OR 'postural stability' OR 'postural control' OR 'posture') | 923 |
| **Cochrane** | (Autism spectrum disorder OR autism) AND (postural balance' OR 'postural stability' OR 'postural control') | 47 |
| **PEDro** | Autism spectrum disorder, motor skills (Simple search) | 22 |
| **Scielo** | Autism spectrum disorder AND balance | 10 |

**Table 2.** Excluded Studies with reasons. *(Online supplementary material)*

| **Authors** | **Title** | **Reason for exclusion** |
| --- | --- | --- |
| Bucci et al.  [55] | The effect of performing a dual task on postural control in children with autism. | Age of participants: 3 to 8 years old |
| Chang et al.  [56] | Visual tasks and postural sway in children with and without autism spectrum disorders. | They use head movements |
| Cheng et al.  [57] | Reactive balance performance and neuromuscular and cognitive responses to unpredictable balance perturbations in children with developmental coordination disorder. | Children with coordination disorder. |
| Chen et al.  [58] | A light fingertip touch reduces postural sway in children with autism spectrum disorders. | They do not provide COP deviation data; they measure the influence of a slight touch on balance. They do not provide baseline data. |
| Chen et al.  [59] | Postural Control and Interceptive Skills in Children with Autism Spectrum Disorder. | It does not provide COP deviation data, and although they apply the MABC, they do not provide data with standard deviations. |
| Chisari et aL[60] | Vestibular Function and Postural Control in Children with Autism Spectrum Disorder | Age of participants: 5 to 12 years old |
| Funahashi et al. [61] | Compensatory postural sway while seated posture during tasks in children with autism spectrum disorder. | They measure anteroposterior and mediolateral COM displacement but in a seated position on a baropodometric. |
| Fournier et al. [62] | Decreased dynamical complexity during quiet stance in children with autism spectrum disorders. | Age of participants: 5.5 (1.1) years old |
| Freitag et al. [63] | Quantitative assessment of neuromotor function in adolescents with high functioning autism and Asperger syndrome. | Age of participants: 14 to 22 years old |
| Gepner et al. [64] | Postural effects of motion vision in young autistic children. | Age of participants: 4 to 7 years old |
| Gouleme et al. [65] | Postural Control and Emotion in Children with Autism Spectrum Disorders. | Postural and oculomotor measurements were taken while the subject explored emotional faces. |
| Kohen-Raz et al. [66] | Postural control in children with autism. | Age of participants: 6-20 years old |
| Li et al. [67] | Automated identification of postural control for children with autism spectrum disorder using a machine learning approach. | Age of participants: 5-12 years old |
| Lim et al. [68] | Effect of Optic Flow on Postural Control in Children and Adults with Autism Spectrum Disorder. | They measure COP in response to visual stimuli and include adults. |
| Lim et al. [69] | Postural control adaptation to optic flow in children and adults with autism spectrum disorder. | They measure COP displacement in response to visual stimuli |
| Mache et al. [70] | Comparison of Postural Control Among College Students with and Without Autism Spectrum Disorder | Age of participants: 18-30 years old |
| Miller et al. [71] | Movement smoothness during dynamic postural control to a static target differs between autistic and neurotypical children. | They analyse spatiotemporal characteristics of the displacement of the centre of mass of the trunk and pelvis using recording cameras. |
| Minshew et al. [72] | Underdevelopment of the postural control system in autism. | Age of participants: 5-52 years old |
| Molloy et al. [73] | Postural stability in children with autism spectrum disorder. | Age of participants: 5-12 years old |
| Perin et al. [74] | Physiological Profile Assessment of Posture in Children and Adolescents with Autism Spectrum Disorder and Typically Developing Peers. | They do not use a validated system to assess balance. |
| Radonovich et al. [75] | Relationship between postural control and restricted, repetitive behaviours in autism spectrum disorders. | Age of participants: 3 to 16 years old |
| Redman-Bentley et al. [76] | Balance and visual skills: Comparison of children with sensory processing disorders and typical development | Participants |
| Siqueiros et al. [77] | Influence of loud auditory noise on postural stability in autistic children: an exploratory study | They measure balance but in response to auditory stimuli. |
| Travers et al. [78] | Motor difficulties in autism spectrum disorder: linking symptom severity and postural stability. | Age of participants: from a few months to 28 years. |
| Tsugita et al. [79] | Fluctuations of the Center of Pressure in Autism Spectrum Disorder | Age of participants: 32.7 ± 7.5 years old |
| Volkan-Yazici et al. [80] | Motor performance and activities of daily living in children with neurodevelopmental disorders | Age of participants: 4.5 to 14.5 years old |
| Yanardag and Yanardag, [81] | Comparative Balance and Gait Analysis Between Preschool Children with Autism Spectrum Disorders and Age-Matched Controls | Age of participants |

**Table 3.** Characteristics of the studies. *(Online supplementary material)*

| **Study** | **Design** | **Sample Size** | **Age (SD) and**  **Gender (%male)** | **BMI, Weight and Hight** | **IQ and**  **ASD degree** | **Assessment tools used and outcome Measures** | **Aim** | **Main Results** |
| --- | --- | --- | --- | --- | --- | --- | --- | --- |
| Abdel Ghafar et al. [29] | Observational cross-sectional comparative study | N=74   - ASD=38. - TD= 36 | **Age:**  ASD= 9.57 (2.08)  TD= 10.84 (2.91)  **Gender**  ASD= 65.79%  TD= 58.33% | **Height (cm).** ASD= 139 (9.72) TD= 131 (5.10).  **Weight (Kg).** ASD= 41.68 (9.13) TD= 37.45 (5.68).  **BMI (Kg/m^2)** ASD= 20.56 (6.41). TD=19.89 (3.05) | **IQ**  ASD= 95 (11).  TD= 115 (9).  **ASD degree**  No information provided | **Balance Assessment:**   - Biodex balance system SD Modified Clinical Test of Sensory Integration and Balance (m-CTSIB).   **Outcome Measures:**   - COP and Overall sway index score | The purpose of this study was to investigate the sensory integration and balance using the Biodex balance system (BBS) in children with ASD during the static posture. | ASD children showed a significant increase in postural sway under all tested conditions when compared to the TD children group, especially for the conditions in which visual and somatosensory inputs were disrupted (*p*-value < 0.05). These results provide evidence that postural stability decreased in ASD children. Under static postural challenges, the current study’s findings imply that children diagnosed with ASD have postural control deficiencies, especially for the conditions in which visual and somatosensory input was disrupted. |
| Ardalan et al. [28] | Observational cross-sectional study Observational cross-sectional comparative study | N= 62   - ASD= 39 - TD= 23 | **Age:**  ASD= 13.08 (3.02)  TD= 14.09 (2.93)  G**ender**  ASD= 95%  TD= 74% | No information provided | **IQ**  ASD= 104.41 (14.95).  TD= 112.39 (9.91)  **ASD degree**  The participants with ASD had a prior clinical diagnosis of ASD  that was supported by meeting criteria for ASD on Modules 3 or 4 of the Autism Diagnostic Observation Scale, 2nd edition (ADOS-2) or by meeting cutoffs on the Social Communication Questionnaire (SCQ)39 and the Social Responsiveness Scale, Revised (SRS-2). participant’s severity of repetitive behaviors and/or restricted interests was measured with the Repetitive Behavior Scale-Revised (RBS-R) but no information provided in the article only they show final results. | **Balance Assessment:**   - BOT-2 - Kinematic data from one-hour training sessions were recorded with a Microsoft Kinect Camera, and postural sway data were recorded with a Wii Balance Board   **Outcome Measures:**   - Kinematic data - Percentil BOT-2 | To assess whether whole-body movement during videogame play can distinguish youth with ASD from those with typical development using machine learning, and to identify key motor features linked to diagnosis and symptom severity. | Youth with ASD showed greater movement variability and instability during gameplay. The classification model reached up to 89% accuracy in distinguishing ASD from typically developing peers. Results were linked to age, motor ability, and autism symptom severity, suggesting a developmental motor delay. Differences were based on full-body movement rather than a specific body part. |
| Biffi et al. 2018 [52] | Observational cross-sectional comparative study | N= 31   - ASD= 15   TD= 16 | **Age:**  ASD= 9.81 (1.57)  TD= 10.01 (1.3)  **Gender:**  ASD= 93.33%  TD= 93.75% | No information provided. | **IQ**  ASD= 99.67 (23.33)  TD= 116.06 (15.09)  **ASD degree**  ADOS (ASD)= 6.87 (1.64)  SRS  ASD= 75.27 (30.21)  TD= 26.38 (12.38) | **Balance Assessment:**  MABC  **Outcome Measures**  MABC- balance | To investigate gait patterns and motor performance in children with autism spectrum disorder compared to typically developing peers, using a protocol that included discrete gait perturbations and standardized motor assessments. | Children with autism showed altered gait patterns, particularly under perturbed conditions, and scored significantly lower on the balance component of the MABC-2. These findings suggest impairments in postural control and adaptability during dynamic motor tasks in this population. |
| Bojanek et al. [30] | Observational cross-sectional comparative study | N=37   - ASD =17 - TD=20 | **Age:**  ASD=13.67 (3).  TD= 12.48 (4.17)  **Gender**  ASD=88%  TD= 80% | **Height (cm).**  ASD= 161.8 (15.88)  TD= 149.46 (17.61).  **Weight (Kg).**  ASD= 58.82 (15.41)  TD= 48.81(18.41).  **BMI (Kg/m^2)**  ASD= 22.18(4.04).  TD=19.71 (4.13) | **FSIQ**   - ASD= 97.76 (17.27) - TD= 108.47 (14.53)   **ASD degree:**  They apply ADI-R and ADOS-2 but no information provided. | **Balance Assessment:**   - Force platform (Model: AccuGait)   **Outcome Measures:**   - COP - Maximum Anticipatory Postural Adjustments (APA) (cm) - APA duration - Body transfer duration - Mean Body transfer velocity - Maximum mediolateral displacement | The present study examined feedback, coordination, and feedforward processes of postural control across static and dynamic standing conditions and stepping to develop a more mechanistic understanding of postural impairments in individuals with ASD. | Individuals with ASD showed reduced MI during circular sway suggesting a reduced ability to effectively coordinate joint movements during dynamic postural adjustments. Additionally, individuals with ASD showed reduced lateral sway when stepping indicating that motor rigidity may interfere with balance and gait. Postural control and stepping deficits were related to repetitive behaviors in individuals with ASD indicating that motor rigidity and key clinical issues in ASD may represent overlapping pathological processes. |
| Bricout et al. [53] | Observational cross-sectional comparative study | N= 42   - ASD= 22 - TD= 20 | **Age:**  ASD= 10.7 (1.3)  TD= 10 (1.6)  **Gender:**  ASD= 100% M. TD= 100% M | **Height (cm).**  ASD= 144.7 (8.7)  TD= 141 (10.5).  **Weight (Kg).**  ASD= 36 (13.3)  TD= 33.3 (7.2).  **BMI (Kg/m^2)** ASD= 16.8 (3.8).  TD= 16.5 (1.5) | **IQ**   - No information provided   **ASD degree:**  Vineland Assesment: Communication:  ASD= 105 (12).  TD= 121 (5). Daily Living skills  ASD= 114 (13).  TD= 132 (12). Socialization: ASD= 88 (11). TD= 106 (9) | **Balance Assessment:**   - M-ABC   **Outcome Measures:**   - VO2 peak - EUROFITT - Motor assesment of Physical and neurological Exam for subtle signs (PANESS) batery. Gait and balance total score: - M-ABC Dexterity (score total of item A, B and C). - Ball skills (score total of items D and E) - Balance (score total of items F, G and H) - Total score of M-ABC | The study objectives are to provide an overall profile of motor capacities in children with ASD compared to neurotypically developed children through specific tests, and to identify which motor tests best discriminate children with or without ASD. | Children with ASD had significantly lower scores in flexibility, explosive power, and strength compared to the control group. Additionally, they exhibited significant difficulties in dexterity and ball skills. Furthermore, children with ASD showed motor skill impairments, with significantly higher PANESS scores for gait, balance, dysrhythmias, and overflow compared to the control group. |
| Bucci et al. [23] | Observational cross-sectional comparative study | N= 46   - ASD= 23. - TD= 23 | **Age:**  ASD= 10.3 (0.4)  TD=10.2 (0.3)  **Gender:**  No information provided | No reported | **IQ**  Wechsler scale (WISC-IV) scores.   - ASD (Verbal comprehension) =101 (6). - ASD (Perceptual reasoning subescale)= 99 (4). - ASD (Working memory)=92 (3). - ASD (Processing speed subscale)= 89(3)   **ASD degree:**  ADI-R scores: Social reciprocal interaction= 11.8 (0.9). Communication=12.2 (0.8). Sterotyped patterns of Behaviors= 5(0.3) ADOS scores: Social reciprocal interaction=8.3 (0.7). Communication=3.9 (0.3) | **Balance Assessment:**   - Multitest Equilibre platform (Framiral^®^). Two viewing conditions (eyes open and eyes closed) were tested on a stable and unstable platform.   **Outcome Measures:**   - Surface of the COP - Mean velocity of the COP - Postural Instability Index | The aim of the study is to compare postural control in children with neuro-developmental disorders such as autism, dyslexia, and hyperactivity with typically developing children. | Children with neurodevelopmental disorder exhibit poor postural control compared to typically developing children, particularly in dynamic environments and when visual information is absent. This may be due to suboptimal cerebellar function and deficiencies in cerebrocortical network. |
| Fears et al. [31] | Observational cross-sectional comparative study | **STUDY 1** N=68  ASD= 27  TD= 41  **STUDY 2 N=53**  ASD= 21  TD= 32 | **Age:**  **STUDY 1**  ASD= 12.44 (3.18)  TD= 10.89 (3.50)  **STUDY 2**  ASD= 12.73 (2.90)  TD= 10.60 (1.94)  **Gender:**  **STUDY 1**  ASD= 81.48%  TD= 39.02%  **STUDY 2**  ASD= 90.48%  TD= 40,63% | Not explicitly reported. An exclusion criterion was a weight of less than 50 lbs (22.68 kg). Height was used to standardize foot position and in the calculation of the sway angle. | **IQ:**  No informed  **ASD degree:**  No informed | **Balance assesment:**  **STUDY 1**  Clinical Test for Sensory Integration in Balance with a portable 20 Hz forcé plate (BioSway, Biodex Corp.) in different conditions  **STUDY 2**  Limits of stability task with of a portable 20 Hz force plate (BioSway, Biodex Corp.)  **Outcome measures of Balance:**  **STUDY 1**   - Sway Magnitude eyes closed and eyes open - Sway Variability eyes closed and eyes open   **STUDY 2**   - Completion time (seconds) - Movement efficiency to different targets. | **STUDY 1**  To examine the age trajectories of static postural stability and sensory reweighting in autistic and neurotypical children, adolescents, and young adults.  **STUDY 2**  To examine the age trajectories of dynamic postural control efficiency in autistic and neurotypical children and adolescents. | **STUDY 1**   - Sway variability decreased as age increased for both groups. - The autistic group showed significantly greater sway variability than the neurotypical group across all conditions, indicating lower overall postural stability. - Both groups showed greater sway magnitude and variability in the Eyes Closed and Dome conditions compared to the Eyes Open condition. - The age trajectory for postural stability was similar between groups, but autistic individuals were consistently less stable.   **STUDY 2**   - Postural control efficiency improved with age for both groups. - ASD participants were significantly less efficient in their movements and took more time to complete the task compared to neurotypical participants - The developmental trajectory for dynamic postural control was similar for both groups, but autistic individuals consistently demonstrated less efficient control. |
| Fournier et al. [24] | Observational cross-sectional comparative study | N= 25  ASD=13.  TD= 12 | **Age:**  ASD= 11.1 (2.3) TD= 12.9 (2.1)  **Gender:**  No informed | **Height (m).**  ASD= 1.45(0.17) TD= 1.57 (0.12).  **Weight (Kg).**  ASD= 50.2 (21.8) TD= 48.2 (10.3). | **Leiter-R Brief IQ.** ASD= 81.8 (32.8).  TD= 104.9 (17.1)  **ASD degree:**  Autism Diagnostic Observation Schedule, Social Communication Questionnaire, or Childhood Autism Rating Scale were used for the diagnostic but no information provided | **Balance Assessment:**  Two adjacent forceplates (Type 4060-10, Bertec  Corp., Columbus, OH).  **Outcome Measures:**   - COP displacement ML and AP - COP Sway - COP displacement in the mediolateral and anteroposterior directions during gait initiation. | The study objectives include investigating postural control in children with ASD during static and dynamic postural challenges, evaluating postural sway during quiet stance and the COP shift mechanism during gait initiation, and assessing the interactions between the COP and COM during quiet stance. | Children with ASD exhibited significantly greater postural sway, larger separations between COP and COM during quiet stance, and intact posterior COP shift mechanism during gait initiation compared to typically developing children. |
| Fradet et al. [46] | Observational cross-sectional comparative study | N=48   - ASD=24 - TD= 24 | **Age:**   - ASD=9.4(1.8) - TD=9.1(1.6)   **Gender:**   - ASD= 62.5% - TD= 41.66% | **Height (cm).**  ASD= 136(13)  TD= 137(11)  **Weight (Kg).**  ASD= 30.5 (11.0)  TD= 29.8 (5.8) | **IQ:**  No informed but they applied Wechsler scale (WISC-V**)**  **ASD degree:**  No informed | **Balance Assessment:**  Bertec.  (60 Å~ 40 cm, FP4060-05-PT-1000, USA), in Limoges a  Kistler. (9260AA), and in Poitiers, a Sensix..(100Hz with 16-bit precisión)  **Outcome Measures:**   - Area (mm2) - RMS AP - RMS ML | The first objective was to provide a more comprehensive analysis of postural control in children with ASD by incorporating a wider range of variables, including linear, nonlinear, and frequency dynamic analysis variables, some of which are related to sensory control and integration. Second, they sought to identify which of these variables were most representative of the specific characteristics of postural control in children with ASD. | The results show that, under conditions most representative of everyday life, the CoP trajectory of children with ASD is more rigid and regular than that of TD children. Furthermore, postural control in children with ASD appears to be more dependent on central processes. These differences may be due to variations in sensory integration abilities and raise questions about whether they persist with age. The greater reliance on central regulation also raises new questions about the ability of children with ASD to maintain postural balance under dual-task conditions. |
| Ghanouni et al. [25] | Observational cross-sectional comparative study (matched case-control design) | N= 51   - ASD=21 - TD= 30 | **Age:**  ASD=11.6 ± 1.5.  TD=11.7 ± 1.8  **Gender:**  No informed | **Height (cm).**  ASD= 1151.6 ± 13.0  TD= 154.7 ± 13.1  **Weight (Kg).**  ASD= 46.3 ± 10.3  TD= 49.7 ± 15.2 | **IQ:**  No informed  (inclusion criteria >80 IQ)  **ASD degree:**  No informed | **Balance Assessment:**  Bertec force plate (type 4060-10, Columbus, OH) was applied to measure the postural sway.  **Outcome Measures:**   - Root mean square (RMS) AP (cm) - RMS ML (cm) - RMS (cm) | Examine the effect of social stimuli vs. object stimuli on postural sway in the boys with high functioning ASD compared to their TD peers | ASD Children showed increased postural sway when viewing social stimuli, such as human faces, compared to non-social stimuli. This effect was not observed in typically developing peers. The findings suggest that social cues may place greater cognitive and sensory demands on children with ASD, affecting their postural stability. Increased sway was particularly evident in the medio-lateral plane and correlated with higher levels of autistic traits. These results highlight the interplay between social processing and motor control in ASD and suggest that postural control may serve as a motor marker of social sensitivity. |
| Gouleme et al. [32] | Observational cross-sectional comparative study | N= 60   - ASD= 30 - TD= 30 | **Age:**  ASD= 12.1 (2.9)  TD= 11.08 (0.5)  **Gender:**  ASD= 86.7%.M  TD= 86.7 %M | No informed | **IQ**  **Wechsler scale (WISC-IV) scores.**  Verbal Comprehension= 96.7 (27.8)  Perceptual Reasoning=91.2 (23.1)  Working memory= 87.5 (20.1).  Processing Speed= 86.2 (19.9)  **ASD degree:**  No informed | **Balance assesment:**   - Movement assesment battery for Children (total impairment score) - Framiral® platform   **Outcome measures of Balance:**   - Surface of CoP (cm2) - Mean velocity of CoP (mm/s) - Romberg Quotient (RQ) | The objective of the study was to explore further postural capabilities in ASD children by measuring spatial as well as temporal displacement of the center of pressure using wavelet analysis | Children with autism spectrum disorder showed significantly greater postural instability compared to typically developing peers across all visual and postural conditions. This was evident in both spatial measures (larger center of pressure area and higher sway velocity) and temporal measures (increased spectral power and reduced cancelling time, particularly at low and medium frequencies). Instability was most pronounced when visual input was removed or perturbed and when somatosensory feedback was compromised. These findings suggest deficits in the integration and use of sensory information, likely linked to cerebellar dysfunction. |
| Graham et al.[33] | Observational cross-sectional comparative study | N=44   - ASD=26 - TD= 18 | **Age:**  ASD=13 (3.2)  TD= 13.4 (1.9)  **Gender:**  ASD: 84,62% TD= 88,89% | **BMI:**  ASD= 21.7 (4.6).  TD= 22.5 (3.6) | The ADOS, ADI-R and DSMIV scales were used but the results are not reported. | **Balance Assessment:**   - Nintendo Wii Balance Board (Nintendo of America Inc., Redmond WA).   **Outcome Measures:**   - COP path lenght in different conditions | The aim of the study is to investigate balance control in individuals with Autism Spectrum Disorder (ASD) and compare it to typically developing (TD) peers. The study also aims to examine the effects of different stance and vision conditions on balance performance in both groups. | Impairments in sensorimotor integration are reported in ASD, and balance deficits in ASD were exacerbated by stance alterations but were not related to symptom severity when age was considered. The single leg stance conditions presented the greatest challenge to the ASD group compared to their typically developing counterparts. Relationships between balance control and symptom severity in ASD were rendered non-significant when age was included in the statistical model, cautioning againsst assuming a direct link between balance impairments and symptom severity. |
| Hannant [34] | Observational cross-sectional comparative study | N= 36   - ASD=18. - TD= 18 | **Age:**   - ASD= 9.93 (2.71). - TD= 9.16 (1.89)   **Gender:**  ASD= 72,22% TD=38,89 % | No information provided | **IQ:**  Non-verbal Reasoning. ASD=90.94 (13.28) TD= 99.5 (12.68)  **ASD degree:**  No information provided | **Balance assesment:**   - MABC-2 - Beery-Buktenica Developmental Test of Visual-Motor Integration, Sixth Edition Visual motor integration.   **Outcome measures of Balance:**   - MABC-2 Balance | The current study explores whether motor coordination and visual perception are impaired in children with ASD, and whether difficulties in coordination and visual perception correlate with receptive language levels. | Results showed that ASD children scored significantly lower on receptive language, coordination and visual motor integration than the TD group. In the TD group receptive language significantly correlated with visual perception; in the ASD group receptive language significantly correlated with balance. These results imply that sensorimotor skills are associated with the understanding of language in ASD and thus the relationship between sensorimotor experiences and language warrants further investigation. |
| Kaur et al. 2017 [49] | Observational cross-sectional comparative study | N= 36   - ASD= 24   (12 HASD and 12 LASD)   - TD= 12 | **Age:**  HASD= 7.44 (0.57)  LASD= 8.74 (0.59)  TD= 7.75  **Gender:**  HASD= 100%  LASD= 83.33% (10:2)  TD= 75% (9:3) | Not reported | **IQ:**  Not specifically reported, but participants had to be able to follow simple instructions (suggesting they were not severely affected).  **ASD degree:**  All children with ASD had a clinical diagnosis based on DSM-IV-TR and the Autism Diagnostic Observation Schedule (ADOS):  HASD= 7.9 (1.76)  LASD= 9.27 (1.10)  **LASD**: Low-functioning Autism Spectrum Disorder  **HASD**: High-functioning Autism Spectrum Disorder | **Balance assesment:**   - Bruininks-Oseretsky Test of Motor Proficiency, 2nd Edition   **Outcome measures of Balance:**   - Standard scores for the BOT-2 Body Coordination composite (which includes the balance subtest). | To compare motor performance (gross and fine motor, praxis/imitation, motor coordination, and interpersonal synchrony) across children with ASD (high IQ and low IQ) and typically developing (TD) children, and to examine the relationship between motor skills, IQ, and ASD severity. | Both ASD groups (HASD and LASD) exhibited lower gross motor scores (including the BOT-2 Body Coordination composite which assesses balance) compared to the TD group. The LASD group had lower Body Coordination scores than the HASD group. Gross motor performance significantly correlated with IQ but not with ASD severity. |
| Li et al. [35] | Observational cross-sectional comparative study | N=22   - ASD=11 - TD=11 | **Age:**  ASD= 8.8 (2.3)  TD= 8.7 (2.3)  **Gender:**  ASD= 90.9 %  TD= 81.81% | **Height (cm).**  ASD= 136 (17)  TD= 133 (15) **Weight (Kg).**  ASD= 33.7 (12.6)  TD=37 (12.2) | **IQ:**  No informed  **ASD degree:**  No informed | **Balance assesment:**  Force plate (60 Hz, type 9286AA, Kistler Instrument Corp., Winterthur, Switzerland)  **Outcome measures of Balance:**   - AP complexity index in different conditions: Eyes open on the stable surface, eyes open on the compliance surface, eyes closed on the stable surface and eyes closed on the compliance - ML complexity index different conditions - Sway area (cm2) different conditions | The purpose of this study was to compare the complexity of postural control between children with autism spectrum disorder (ASD) and typical developing children during altered visual and somatosensory conditions using the multiscale entropy. | Children with ASD exhibited lower complexity in mediolateral sway compared to typical developing children, while anteroposterior sway complexity did not show a similar decrease. The altered visual or somatosensory conditions did not significantly affect postural sway complexity, leading to the conclusion that the complexity of postural control for children with ASD was partially compromised. |
| Lidstone et al. [36] | Observational cross-sectional comparative study | N=45   - ASD=23. - TD= 22 | **Age:**  ASD= 12.4 (2.8).  TD= 11.7 (2.7)  **Gender:**  ASD= 83%  TD=64% | **Height (m)** ASD=1.50 (0.1)  TD= 149 (9).  **Body mass (kg).**  ASD=53.4 (22.5)  TD= 47.6 (17) | **FSIQ-2**  ASD= 93 (+-13).  TD=112 (+-10)  **ASD degree:**  No informed | **Balance assesment:**  Portable force plate (model BP5050) during 30-second bilateral quiet stance trials, and a unipedal stance test was used to evaluate static balance.  **Outcome measures of Balance:**   - COP - Sway area, mediolateral and anteroposterior - Sway magnitude - Velocity - Sample entropy. - Static balance was assessed via unipedal stance time. | To identify postural control features that are uniquely impaired in children with Autism Spectrum Disorder (ASD) by comparing them with children with ADHD, Fetal Alcohol Spectrum Disorder (FASD), and typically developing peers. | Children with ASD showed significantly greater postural sway area and mediolateral sway magnitude and velocity than all other groups. In contrast, anteroposterior sway differences were only significant when compared to typically developing children. Static balance, as measured by unipedal stance time, was reduced across all clinical groups compared to controls, but only children with ASD exhibited ASD-specific postural control impairments. |
| Lim et al. [37] | Observational cross-sectional comparative study | N=33   - ASD= 15. - TD= 18 | **Age:**  ASD= 9.7 (1.3). TD= 10.0 (1.3)  **Gender:**  ASD= 80%  TD= 66.67% | **Height (cm)** ASD=142.7 (9.3)  TD= 143.4 (8.7). **Weight (kg).** ASD=35.6 (9.1)  TD= 35.7 (6.6) | **IQ:**  **WASI**  ASD=110.2 (21.1)  TD= 119.0(9.7)  **ASD degree:**  SRS (Social Responsiveness Scale)  ASD= 80.7 (8.2)  TD= 48.2 (7.2) | **Balance assesment:**  Force platform (AccuGait, AMTI)  **Outcome measures of Balance:**   - Mean anteroposterior and mediolateral position of the center of pressure (COP) - Root mean square (RMS) of COP in both directions - COP velocity - Sample entropy (as a proxy for attentional investment in postural control) | To examine whether children with autism spectrum disorder (ASD) differ from typically developing children in postural stability and attentional demands during quiet standing, under conditions with and without visual input. | Children with ASD and typically developing children showed similar postural stability and attentional demands across both visual conditions. Both groups demonstrated increased sway and higher attentional demand when visual input was removed. The findings suggest that visual information supports postural control in both groups, and that basic postural control mechanisms may not differ significantly in children with and without ASD before adolescence. |
| Mache and Todd [38] | Observational cross-sectional comparative study | N=22   - ASD= 11 - TD= 11 | **Age:**  ASD= 9.46 (2.5).  TD= 9.35 (2.41)  **Gender:**  ASD= 90.01 % (10 M 1 F)  TD= 81.81% M (9M 2F) | No informed | **IQ:**  No informed  **ASD degree:**  Repetitive Behavior Scale-Revised: | **Balance assesment:**   - Test of Gross Motor Development-3rd Edition (TGMD-3) - Ground reaction force (GRF) data at 60 Hz using a single force plate.   **Outcome measures of Balance:**   - TGMD-3 - Sway área solid surface. - Sway área compliant surface | To examine the relationship between gross motor skills, postural stability, and restricted/repetitive behaviors in children with and without ASD. | Children with ASD exhibited significantly greater postural sway and lower gross motor scores (TGMD-3) compared to typically developing peers. Sway area on a solid surface, diagnosis, and age significantly predicted gross motor performance. Repetitive behaviors did not correlate with motor skill outcomes. |
| Martín-Díaz et al. [14] | Observational cross-sectional comparative study | N=100  ASD= 50  TD= 50 | **Age:**  ASD= 9.54 (3.09)  TD= 9.54 (3.09)  **Gender:**  ASD= 86%  TD= 86% | No informed | No informed | **Balance assessment:**   - PBS - TUG - SFBOT-2   **Outcome measures of Balance:**   - PBS - TUG - SFBOT-2 | Analyse the differences in static and dynamic balance, postural control, and motor skills in children and adolescents with ASD with respect to children and adolescents with TD using balance and motor skills assessment scales and tests. | Children with ASD present difficulties in motor skills and in static and dynamic balance compared to children with NTD. Differences were observed in the motor skills of strength followed by manual dexterity, running speed and agility, fine motor precision, fine motor integration, and balance. The PBS item that showed the greatest difference between the ASD group and control group was maintaining monopodial support with hands on hips. Finally, poor to moderate correlations were obtained between the different tests with statistically significant differences. |
| Memari et al. (2013) [40] | Observational cross-sectional comparative study | N= 52   - ASD= 21. - TD= 30 | **Age:**  ASD= 11.5 (1.6).  TD= 11.6 81.9)  **Gender:**  ASD=100%  TD= 100% | **Height (cm)** ASD=151.2 (12.9)  TD= 153.8 (13.7)  **Weight (kg).**  ASD= 45.6 (10.3)  TD= 48.9 (15.5) | **Non verbal IQ:**  ASD= 93.3 (11.1)  TD= 98.1 (8.7)  **ASD degree:**  Score on severity of autistic symptoms (max. 179)/ATEC:   - ASD= 78.6 (11.9)   Score on rate of autistic symptoms (max. 80)/AQ:   - TD= 20.2 (4) | **Balance assesment:**  Bertec force plate (type 4060-10, Columbus, OH).  **Outcome measures of Balance:**  Postural  sway parameters: root mean square (RMS) in AP, ML and composite measures, mean velocity in AP, ML and composite  measures, range in AP and ML directions, mean frequency and sway área. | To compare postural sway parameters in children with autism spectrum disorder (ASD) and typically developing (TD) children, and to examine how sway is influenced by autism severity and age. | Children with ASD showed significantly higher postural instability than in TD peers, with greater sway in both anteroposterior and mediolateral directions. They also demonstrated lower sway frequency and a stronger association between sway magnitude and autism severity. Postural control impairments in the ASD group were more pronounced in the mediolateral axis, and unlike in TD children, sway did not correlate with age, height, or weight. |
| Memari et al. (2014) [39] | Observational cross-sectional comparative study | N= 35   - ASD=20 - TD= 15 | **Age:**  ASD=11.9 (1.6).  TD= 11.8 (1.7)  **Gender:**  ASD= 100%.  TD= 100% | No informed | **IQ non-verbal** ASD= 94.3 (9.1) TD= 97.6 (7.6)  **ASD degree:**  The diagnosis of ASD was based on both DSM-IV and the autism diagnostic inventory-revised (ADI-R) by a child psychiatrist. Results no informed | **Balance assesment:**  Bertec force plate (Model 4060-10, Columbus, OH) at 200 Hz.  **Outcome measures of Balance:**   - RMS (root mean square) AP(cm) - RMS ML (cm) - RMS (cm) - Mean velocity AP (cm/s) - Mean velocity ML (cm/s) - Mean velocity (cm/s) - Sway area (cm2/s) | Compare the interference of visual or auditory tasks on postural control in children with autism spectrum disorder (ASD) compared to typically developing children. | Children with ASD showed higher postural sway scores in visual tasks compared to auditory tasks, and they exhibited significantly higher sway scores in all parameters compared to typically developing children. The study indicates that visual and auditory tasks may differently influence postural control in children with ASD. |
| Miller et al. [41] | Observational cross-sectional comparative study | N=18   - ASD= 10 - TD= 8 | **Age:**  ASD= 13 (2.54)  TD= 10.2 (3.41)  **Gender:**  ASD= 90% M (10 M, 1F).  TD= 50% (4M, 4F) | No informed | **IQ**  ASD= 98.3.  TD= 114.13  **ASD degree:**  ASD and DCD participants carried a prior diagnosis based on DSM-IV or DSM-V criteria and these were confirmed by the reseaºrch team. | **Balance assesment:**  Force plate system integrated into a virtual environment  **Outcome measures of Balance:**   - COP velocity AP and ML - COP acceleration AP and ML | Compared acceleration and velocity of center of pressure in  ASD and Developmental Coordination Disorder (DCD) and typical development children | ASD and DCD share similar difficulty with motor control. This study found differences in acceleration profiles between ASD, DCD, and TD. Children with DCD had less fluid movement than children with ASD or TD. Characteristics of dynamic postural control may inform more targeted interventions. |
| Pettinato et al. [27] | Observational cross-sectional comparative feasibility study | N=22   - ASD=11 - TD=11 | **Age:**  ASD= 12 (1.7)  TD= 12.8 (1.4)  **Gender**  No informed | **Height (cm)**  ASD= 159.4 (6.7)  TD= 161.7 (8.9)  **Weight (kg)**  ASD= 57.2 (9.5)  TD=55.9 (10.2) | **IQ:**  ASD= 90.4 (17.7)  TD=96.5 (10.8)  **ASD degree:**  ADOS total score  ASD= 10. 6 (4.1)  TD= 0.4 (0.7) | **Balance assesment:**  Force platform (Kistler 9286 B, Winterthur, Switzerland; 100 Hz sampling frequency) and the signals were sent to the SMART-D system (BTS, Garbagnate Milanese, MI, IT) for offline processing.  **Outcome measures of Balance:**   - COP oscillations - RMS - Sway path - Sway area | To investigate whether combining linear and non-linear parameters of the COP during stance in subjects with ASD, could provide insight on specific features of motor dysfunction possibly linked to ASD cognition and clinical characteristics. | Compared to controls, subjects with ASD showed reduced postural stability and complexity, with higher regularity of COP trajectories, particularly in the most unstable feet positions, during visually guided saccades and in the medial-lateral direction. Spearman correlations indicated that, in the patients’ group, postural instability was associated with a decrease in the geometric complexity and an increase in the regularity of the COP trajectory. Moreover, the increase in regularity of the COP trajectory was associated to the severity of restricted and repetitive behavior. |
| Shabana et al. [12] | Observational cross-sectional comparative study | N=35   - ASD=20 - TD= 15 | **Age:**  ASD= 8.95 (2.98)  TD= 9 (1.6)  **Gender:**  ASD= 65%  TD= 60% | No informed | **IQ:**   - No informed   **ASD degree:**   - No informed | **Balance assesment:**  Computerized Dynamic Posturography (CDP) via the Sensory Organization Test (SOT) on the SMART Equitest system.  **Outcome measures of Balance:**  Sensory Organization Test | To assess postural control in children with autism and examine its correlation with age and autism severity as measured by the Child Autism Rating Scale (CARS). | Children with autism had significantly lower SOT scores compared to controls, particularly when somatosensory input was disrupted. There was a positive correlation between age and postural stability, and a negative correlation between CARS scores and balance performance, indicating that greater autism severity was associated with poorer postural control. |
| Smoot Reinert et al. [26] | Quasi-experimental pre-post feasibility study | N=10   - ASD= 5 - TD= 5 | **Age:**   - ASD= 9.2 ± 0.5 - TD= 7.4 ± 2.1   **Gender:**  No informed | **Height (m).**  ASD= 1.39 ± 0.07  TD= 1.28 ± 0.13  **Weight (Kg).**  ASD= 37.1 ± 9.5  TD= 28.0 ± 8.3 | **IQ:**   - No informed   **ASD degree:**   - No informed | **Balance Assessment:**   - Functional Reach score (inches) - BOT-2 - Force measuring platform (Model BP505, Bertec Corp., Worthington, OH)   **Outcome Measures:**   - Sway anteroposterior and medio lateral - Sway Velocity - RMS | To examine the feasibility of using posturography to measure immediate changes in postural control in children with ASD following vestibular-based sensory integration (SI) therapy using a swing. | Children with ASD showed greater postural sway and lower sample entropy than typically developing peers at baseline. After the intervention, most children with ASD showed reduced sway velocity and lower anterior-posterior sample entropy, suggesting improved postural control. Posturography detected short-term physiological changes following vestibular input. |
| Somogyi et al. [48] | Quasi-experimental pre-post study | N= 30   - ASD= 18. - TD= 12 | **Age:**  ASD=7.83  TD=8.08  **Gender:**  ASD=77.78%  TD= 66.67% | No informed | **Nonverbal IQ**.  ASD= 98 (17.9)  TD= 124 (7.3)  **ASD degree:**  **Receptive language (PPVT-R)**.  ASD= 81 (20).  **ASD symptom severity (CARS).**  ASD= 35 (2.23) | **Balance assesment:**  Virtual Human Interface platform© (Digital Elite/PanoCAST, Inc., Los Angeles, CA) Nintendo Wii balance board  **Outcome measures of Balance:**   - Sway área - Sway length   Both measures were recorded under two experimental conditions: baseline condition and Visual Feedback Condition. | To assess baseline postural stability in children with ASD under 12 years of age and to examine whether visual feedback improves their balance control. | Children with ASD showed significantly poorer postural stability than typically developing peers. When given visual feedback, their postural control improved notably, although it remained lower than in the control group. The effect of visual feedback was more pronounced in children with lower IQ scores. |
| Stania et al. [26] | Observational cross-sectional comparative study (case-control design) | N=32   - ASD= 16. - TD= 16 | **Age:**  ASD= 8.13 (1.54).  TD= 7.93 (0.88)  **Gender:**  ASD= 68%  TD= 56.25% | **Height (cm).**  ASD= 133.06 ± 10.62.  TD= 130.62 ± 8.07  **Weight (Kg).**  ASD= 29.98 ± 9.32  TD=27.39 ± 6.41 | **IQ:**  No informed  **ASD degree:**  They used ADOS-2 to identified ASD participant but they do no informed about results | **Balance Assessment:**  Force platform (AMTI, AccuGait, Watertown, MA, USA)  **Outcome Measures:**  COP sway range (ra), root mean  square (rms) as well as rambling and trembling components in  antero-posterior and medio-lateral directions | The main aim of the study was to assess postural control in autistic children using advanced analytical methods of COP measurements, including rambling-trembling signal decomposition and sample entropy. | Compared to typically developing children, those with autism spectrum had significantly higher values of COP and rambling trajectory parameters in the antero-posterior direction during quiet standing. The variables of the trembling trajectory did not differ significantly between the groups. The autistic children had significantly lower values of sample entropy in the antero-posterior direction compared to typically developing children. |
| Stania et al. [47] | Observational cross-sectional comparative study (case-control design) | N= 32   - ASD= 16 - TD= 16 | **Age**  TD= 7.93 (0.88)  ASD=8.13 (1.54)  **Gender (%male):**  TD=56.25%  ASD) = 68.75% | **Height (cm)**  TD= 130.62 (8.07)  ASD= 133.06 (10.62)  **Weight (kg)**  TD= 27.39 (6.41)  ASD= 29.98 (9.32) | **IQ:**   - No informed   **ASD degree:**   - No informed | **Balance assessment:**  Force platforms sized 502 mm (length) / 502 mm (width) / 45 mm (height) (AMTI, AccuGait, Watertown, MA, USA), 100Hz  **Outcome measures of Balance:**   - COP AP (cm) - COP ML (cm) - Velocity COP AP (cm/s) - Velocity COP ML (cm/s) | To assess postural control during quiet standing before and after step initiation tasks in autistic children. | Autistic children show increased postural sway before and after transitional locomotor tasks compared to typically developing children. |
| Stins et al. [49] | Observational cross-sectional comparative study | N=18   - ASD= 9 - TD= 9 | **Age:**  ASD= 10.8 (1.2)  TD= 10.8 (1.2)  **Gender (% male):**  ASD= 88.89%  TD= 88.89%. | **Height (cm)** ASD=150 (13)  TD= 149 (9).  **Weight (kg).** ASD=41.3 (13.3)  TD= 36.7 (7.75) | **IQ:**   - No informed   **ASD degree:**   - No informed | **Balance assessment:**   - Movement ABC - Nintendo© Wii Balance Board that collected postural data   **Outcome measures of Balance:**   - Standard deviation of sway, separate for the anterio-posterior (AP) and mediolateral (ML) direction. - COPRANGE-ML and COPRANGE-AP, i.e., the distance between the maximal postural excursions in the mediolateral direction and the anterio-posterior direction, respectively - Sway path length (SPL), i.e., the summed length of the postural excursions in the AP–ML plane over the measurement interval. | The study objectives are to compare postural tasks between children with mild ASD and controls, test the effects of sensory and cognitive disturbances on quiet standing, and examine the impact of cognitive and sensory disturbances on balance and postural control in children with mild autism. | The main findings of the study suggest that individuals with ASD have a greater destabilizing effect of closing the eyes and a tighter coupling between vision and motor adjustments compared to controls. The study found evidence of abnormal postural control in children with mild ASD, with mild effects on the attentional regulation of balance in the ASD sample. |
| Surgent et al. [43] | Randomized controlled trial | N=62   - ASD=34 - TD=28 | **Age:**  Autistic balance training (n = 17) = 15.6 (1.27).  Autistic control (n=17) = 15.44 (1.35).  Non-autistic balance training (n=15) =15.08 (1.42).  Nonautistic control (n=13) = 14.87 (1.59)  **Gender:**  Autistic balance training (*n* = 17) = 94%.  Autistic control (n=17) = 88%  Non-autistic balance training (n=15) =73% M  Non autistic control (n=13)= 85% M | **BMI (Kg/m^2)**  **ASD**  Autistic balance training (*n* = 17) = 27.11 (7.13)  Autistic control (n=17) =24.66 (5.54)  **TD.**  Non-autistic balance training (n=15) =22.03 (4.67)  Nonautistic control (n=13) = 21.38 (2.54) | **FSIQ MEAN**  **ASD.**  Autistic balance training (*n* = 17) = 106.06(18.09)  Autistic control (n=17) =101.82 (17.36)  **TD.**  Non-autistic balance training (n=15) =110. 73 (9.64)  Nonautistic control (n=13) = 117.46 (14.67).  **Performance IQ**  **ASD.**  Autistic balance training (*n* = 17) = 106.59 (20.33)  Autistic control (n=17)=106.18 (18.37)  **TD.**  Non-autistic balance training (n=15)=109.4 (9.36)  Non autistic control (n=13)= 123.92 (20.50)  **Verbal IQ.**  **ASD.**  Autistic balance training (*n* = 17)= 104.76 (18.45)  Autistic control (n=17)=97.47 (17.9)  **TD.**  Non-autistic balance training (n=15)=109.33 (12.52).  Non autistic control (n=13)= 109.46 (14.50) | **Balance assesment:**  Wii  Balance Board  **Outcome measures of Balance:**  COP  Postural Sway área  Balance time | The present RCT had three objectives. The first objective was to rigorously test whether biofeedback-based balance training improved balance in individuals with ASD. The second objective of the study was to identify which brain structures would demonstrate changes as a result of the balance training. The third and final aim was to examine whether balance training improved core autism symptoms and daily living skills. | Biofeedback-based balance training significantly improved balance and decreased parent-reported autism symptom severity. However, no training-related changes to daily living skills were observed, nor did we observe hypothesized changes in the microstructure of the corticospinal tract. Instead, they found a wide range of balance-related structural changes across the brain, and these changes were often distinct in the autistic participants compared to the non-autistic participants. This finding suggests distinct microstructural changes in response to balance training in autistic individuals which may be indicative of distinct neural substrates of balance in ASD. |
| Travers et al. [11] | Observational cross-sectional comparative study | N= 37   - ASD= 21 - TD= 16 | **Age:**   - ASD= 9.63 (2.09) - TD= 9.64 (2.78)   **Gender:**   - ASD= 85.7%. - TD= 87.5% | **BMI**  ASD= 18.03 (4.79)  TD= 17.75 (4.2) | **FSIQ mean**  ASD= 102.2 (12.85).  TD= 117.56 (15.15).  **Performance IQ**  ASD= 104.8 (17.91)  TD= 110.5 (16.77)  **Verbal IQ.**  ASD= 99.05 (12.2)  TD= 120.5 (14.39).  **ASD degree**  Repetitive Behavior Scale-Revised. total raw  ASD= 29.36 (21.33).  Social Responsiveness Scale total raw  ASD= 87.28 (23.11) | **Balance assesment:**  Wii balance board  **Outcome measures of Balance:**  Sway area (mm2) | Examine postural stability in children and adolescents on the autism spectrum compared to children and adolescents with typical development during standing on a traditional fixed platform compared to a tiltable platform. | The results demonstrated that youth on the spectrum who had lower VIQ exhibited greater balance challenges regardless of condition, and youth on the spectrum who had lower IQ exhibited more balance challenge in the unsteady surface condition. Therefore, IQ should be taken into account when determining whether a youth on the autism spectrum might have difficulty maintaining balance in more true-to-life settings where balance is required. |
| Wang et al. [44] | Observational cross-sectional comparative study | N= 43   - ASD=22. - TD= 21 | **Age:**  ASD= 12.72 (3.64)  TD= 11.67 (4.53)  **Gender:**  SD= 86.4%. TD= 85.7% | **FSIQ mean.**  ASD= 98.68.  TD= 108.05 (14.29).  **Performance IQ.**  ASD= 103.45 (16.74).  TD= 104.24 (12,69).  **Verbal IQ.**  ASD=94.5 (18.01).  TD= 109.81 (15.26)  **ASD degree:**  Were required to have a score of 8 or lower on the Social Communication Questionnaire (SCQ) but result no informed | **Height (cm).**  ASD= 154.3 (24.45)  TD= 142.9 (23.09).  **Weight (Kg).**  ASD=55 (27.54)  TD= 41.77 (20.88) | **Balance assesment:**  AccuGait strain gauge force platform  (size 49.78 × 49.78 cm; sampling rate of 1000 Hz).  **Outcome measures of Balance:** | The study had three aims: (1) to quantify the extent to which children with ASD showed increased postural sway during static and dynamic stances, (2) to quantify postural orientation processes in ASD by characterizing spatial and temporal dimensions of their postural sway relative to their own postural limitation boundary, and (3) to quantify postural equilibrium processes in ASD by determining the amount of shared COPAP and COPML information during quiet and dynamic standing postures. | Children with autism spectrum disorder exhibited greater variability and longer trajectories in their center of pressure across all standing conditions, with postural instability more evident during dynamic tasks. In these dynamic situations, they also showed reduced spatial and temporal margins for maintaining balance, as reflected by lower values in the directional distribution and timing estimates of the center of pressure approaching the limits of stability. This suggests difficulties in processing spatial information and in anticipating the postural adjustments needed to prevent imbalance. Furthermore, increased coordination between movements in the forward–backward and side-to-side directions indicated reduced independence between ankle- and hip-based control strategies. These changes in postural control were also linked to higher levels of repetitive behaviors, emphasizing their clinical importance. |
| Whyatt et al. [51] | Observational cross-sectional comparative study | N= 59  ASD=18  Receptive vocabulary control= 19  Non verbal IQ control= 22 | **Age:**  ASD= 10.03 (1.2)  Receptive vocabulary control (TD)= 10.99 (3.3).  Non-verbal IQ control (TD)= 9.36 (1.2)  **Gender:**  ASD= 61.11%  Receptive vocabulary control (TD)= 31.58%  Non-verbal IQ control (TD)= 50% | **IQ:**  No informed  **ASD degree:**  No informed | **Height (cm).**  No informed  **Weight (Kg).**  No informed | **Balance assesment:**  MABC-2  **Outcome measures:**   - Manual dexterity - Ball skills - Balance - Total | The present study thus sets out to provide a clear picture of motor ability specific to autism by examining the motor skills of children with autism compared to two groups of typically developing children; (1) a language (receptive vocabulary) matched group and (2) a nonverbal IQ matched group. | The overall standardised M-ABC2 results suggest that children with ASD experience a level of general motor impairment when compared to typically developing children matched on measures of either receptive vocabulary or non-verbal IQ. |
| Zoccante et al. [45] | Observational cross-sectional comparative study (case-control design) | N=43   - ASD=20 - TD= 23 | **Age:**  ASD= 10.7 (2.0) TD=12.3 (2.6)  **Gender:**  ASD= 80% M. TD= 43,5% M | **IQ:**  ASD= 94.1 (15.9) TD=105.2 (15.5)  **ASD degree:**  No information provided | No information provided | **Balance Assessment:**  Sensory Organization Test | The aim of the study was to investigate whether postural control deficits vary across neurodevelopmental conditions, specifically ASD, attention deficit hyperactivity disorder (ADHD), and Tourette syndrome (TS), compared to typically developing children. The study also sought to test the neurodevelopmental gradient hypothesis, which proposes that neurodevelopmental disorders exist on a continuum of impairment severity, with ASD representing the most severe form. | Children and adolescents with ASD showed significantly poorer postural stability across multiple sensory conditions and balance parameters when compared to typically developing peers. ADHD participants exhibited deficits only under the most challenging sensory condition, while children with Tourette syndrome performed similarly to controls. Comparisons among clinical groups confirmed that postural control impairments were most severe in ASD, followed by ADHD, with TS showing the least impairment. These findings support the neurodevelopmental gradient hypothesis and highlight poor postural control as a potential marker of neurodevelopmental severity. |

**Table 4.** Critical Review (Online supplementary material)

|  | **1** | **2** | **3** | **4** | **5** | **6** | **7** | **8** | **9** | **10** | **11** | **12** | **13** | **14** | **15** | **TOTAL*** |
| --- | --- | --- | --- | --- | --- | --- | --- | --- | --- | --- | --- | --- | --- | --- | --- | --- |
| A  bdel Ghafar et al. 2022 | 1 | 1 | 1 | 1 | 1 | 1 | 1 | Not addressed | N/A | N/A | 1 | 1 | 1 | 0 | 1 | 11/13 |
| Ardalan et al. 2019 | 1 | 1 | 1 | 1 | 0 | 1 | 1 | Not addressed | N/A | N/A | 1 | 1 | 0 | 0 | 1 | 9/13 |
| Biffi et al. 2018 | 1 | 1 | 1 | 1 | 0 | 1 | 1 | Not addressed | N/A | N/A | 1 | 1 | 0 | 0 | 1 | 9/13 |
| Bojanek et al. 2020 | 1 | 1 | 1 | 1 | 0 | 1 | 1 | Not addressed | N/A | N/A | 1 | 1 | 0 | 0 | 1 | 9/13 |
| Bricout et al. 2019 | 1 | 1 | 1 | 1 | 0 | 1 | 1 | Not addressed | N/A | N/A | 1 | 1 | 0 | 1 | 1 | 10/13 |
| Bucci et al. 2017 | 1 | 1 | 1 | 1 | 0 | 1 | 1 | Not addressed | N/A | N/A | 1 | 1 | 0 | 0 | 1 | 9/13 |
| Fears et al. 2022 | 1 | 1 | 1 | 1 | 0 | 1 | 1 | Not addressed | N/A | N/A | 1 | 1 | 0 | 0 | 1 | 9/13 |
| Fournier et al. 2010 | 1 | 1 | 1 | 1 | 0 | 1 | 1 | Not addressed | N/A | N/A | 1 | 1 | 0 | 1 | 1 | 10/13 |
| Fradet et al. 2025 | 1 | 1 | 1 | 1 | 1 | 1 | 1 | Not addressed | N/A | N/A | 1 | 1 | 0 | 0 | 1 | 10/13 |
| Ghanouni et al. 2017 | 1 | 1 | 1 | 1 | 1 | 1 | 1 | Not addressed | N/A | N/A | 1 | 1 | 0 | 1 | 1 | 11/13 |
| Gouleme et al. 2017 | 1 | 1 | 1 | 1 | 0 | 1 | 1 | Not addressed | N/A | N/A | 1 | 1 | 0 | 0 | 1 | 9/13 |
| Graham et al. 2015 | 1 | 1 | 1 | 1 | 0 | 1 | 1 | Not addressed | N/A | N/A | 1 | 1 | 0 | 0 | 1 | 9/13 |
| Hannant 2018 | 1 | 1 | 1 | 1 | 0 | 1 | 1 | Not addressed | N/A | N/A | 1 | 1 | 0 | 0 | 1 | 9/13 |
| Kaur et al. 2017 | 1 | 1 | 1 | 1 | 0 | 1 | 1 | Not addressed | N/A | N/A | 1 | 1 | 0 | 0 | 1 | 9/13 |
| Li et al. 2019 | 1 | 1 | 1 | 1 | 1 | 1 | 1 | Not addressed | N/A | N/A | 1 | 1 | 0 | 0 | 1 | 10/13 |
| Lidstone et al. 2020 | 1 | 1 | 1 | 1 | 0 | 1 | 1 | Not addressed | N/A | N/A | 1 | 1 | 0 | 0 | 1 | 9/13 |
| Lim et al. 2020 | 1 | 1 | 1 | 1 | 0 | 1 | 1 | Not addressed | N/A | N/A | 1 | 1 | 0 | 0 | 1 | 9/13 |
| Mache and Todd, 2016 | 1 | 1 | 1 | 1 | 0 | 1 | 1 | Not addressed | N/A | N/A | 1 | 1 | 0 | 0 | 1 | 9/13 |
| Martín-Díaz et al. 2024 | 1 | 1 | 1 | 1 | 1 | 1 | 1 | Not addressed | N/A | N/A | 1 | 1 | 0 | 0 | 1 | 10/13 |
| Memari et al. 2013 | 1 | 1 | 1 | 1 | 0 | 1 | 1 | Not addressed | N/A | N/A | 1 | 1 | 0 | 0 | 1 | 9/13 |
| Memari et al. 2014 | 1 | 1 | 1 | 1 | 0 | 1 | 1 | Not addressed | N/A | N/A | 1 | 1 | 0 | 0 | 1 | 9/13 |
| Miller et al. 2019 | 1 | 1 | 1 | 1 | 0 | 1 | 1 | Not addressed | N/A | N/A | 1 | 1 | 0 | 0 | 1 | 9/13 |
| Pettinato et al. 2024 | 1 | 1 | 1 | 1 | 0 | 1 | 1 | Not addressed | N/A | N/A | 1 | 1 | 0 | 0 | 1 | 9/13 |
| Shabana et al. 2012 | 1 | 1 | 1 | 1 | 0 | 1 | 1 | Not addressed | N/A | N/A | 1 | 1 | 0 | 0 | 1 | 9/13 |
| Smoot Reinert et al. 2015 | 1 | 1 | 1 | 1 | 0 | 1 | 1 | 1 | N/A | Not addressed | 1 | 0 | 0 | 0 | 1 | 9/14 |
| Somogyi et al. 2016 | 1 | 1 | 1 | 1 | 0 | 1 | 1 | 1 | N/A | Not addressed | 1 | 1 | 0 | 0 | 1 | 10/14 |
| Stania et al. 2023 | 1 | 1 | 1 | 1 | 1 | 1 | 1 | Not addressed | N/A | N/A | 1 | 1 | 0 | 0 | 1 | 10/13 |
| Stania et al. 2024 | 1 | 1 | 1 | 1 | 1 | 1 | 1 | Not addressed | N/A | N/A | 1 | 1 | 0 | 0 | 1 | 10/13 |
| Stins et al. 2015 | 1 | 1 | 1 | 1 | 0 | 1 | 1 | Not addressed | N/A | N/A | 1 | 1 | 0 | 0 | 1 | 9/13 |
| Surgent et al. 2021 | 1 | 1 | 1 | 1 | 1 | 1 | 1 | 1 | N/A | 1 | 1 | 1 | 1 | 0 | 1 | 13/14 |
| Travers et al. 2018 | 1 | 1 | 1 | 1 | 0 | 1 | 1 | Not addressed | N/A | / N A | 1 | 0 | 0 | 1 | 1 | 9/13 |
| Wang et al. 2016 | 1 | 1 | 1 | 1 | 0 | 1 | 1 | Not addressed | N/A | N/A | 1 | 1 | 0 | 0 | 1 | 9/13 |
| Whyatt and Craig, 2012 | 1 | 1 | 1 | 1 | 1 | 1 | 1 | Not addressed | N/A | N/A | 1 | 1 | 0 | 0 | 1 | 10/13 |
| Zoccante et al. 2021 | 1 | 1 | 1 | 1 | 0 | 1 | 1 | Not addressed | N/A | N/A | 1 | 1 | 0 | 0 | 1 | 9/13 |

0 = not fulfilled criterion; 1 = fulfilled criterion; N/A = not applicable.  Item 1: study purpose; item 2: literature review; item 3: study design; item 4: sample description; item 5: sample size; item 6: reliability of outcomes; item 7: validity of outcomes; item 8: intervention description; item 9: contamination; item 10: cointervention; item 11: statistical significance; item 12: statistical analysis; item 13: clinical importance; item 14: drop-outs reported; item 15: conclusions.

***** Items marked as N/A (Not Applicable) are excluded from the denominator in the final score calculation, as they do not apply to the study design being assessed. Only items rated as Yes, No, or Not addressed are counted toward the total number of applicable items.

**Table 5.** GRADE scale. *(Online supplementary material)*

| **Number of studies** | **Risk of Bias** | **Inconsistency†** | **Indirectness‡** | **Imprecision§** | **Publication Bias¶** | **SMD (95% CI)** | **Quality of Evidence** |
| --- | --- | --- | --- | --- | --- | --- | --- |
| **Balance Performance in ASD vs. TD Children** | | | | | | | |
| *Assessment Using the MABC Balance* | | | | | | | |
| Four trials (n=168)  [24, 27, 31, 46] | Serious | No Serious (I^2^= 37%) | No serious | No serious | No serious | 0.66 (-1.07, -0.25) | Low |
| *Assessment Using Force Platforms - Mediolateral Displacement of the COM* | | | | | | | |
| Five trials (n ≈ 346)  [23,39,41,43, 44] | Serious | Serious (I^2^= 54%) | No serious | No serious | Likely | 0.83 (0.45, 1.21) (EO) / 0.56 (0.09, 1.03) (EC) | Very Low |
| *Assessment Using Force Platforms - Anteroposterior Displacement of the COM* | | | | | | | |
| Five trials (n ≈ 323)  [23,39, 41, 43, 44] | Serious | Very Serious (I^2^= 77%) | No serious | No serious | Likely | 0.97 (0.39, 1.56) (EO) | Very Low |
| *Assessment Using Force Platforms - COM Displacement Area* | | | | | | | |
| Six trials (n = 321–128, depending on condition)  [12,25,37,40,49,51] | Serious | Very Serious (I² = 94–98%) | No serious | Serious (unstable sensitivity analysis) | Likely | 1.15 to 7.72 (depending on condition) | Very Low |
| *Assessment Using Force Platforms - Velocity of COM Displacement* | | | | | | | |
| Four trials (n=190)  [25, 35, 39, 41] | Serious | Serious to Very Serious (I² = 0–85%) | No serious | No serious | Likely | 1.00 to 3.23 (depending on condition) | Low |
